# Transcriptome analysis of *Thevetia peruviana* cell suspensions treated with methyl jasmonate reveals genes involved in phenolics, flavonoids and cardiac glycosides biosynthesis

**DOI:** 10.3389/fpls.2025.1593315

**Published:** 2025-05-26

**Authors:** Olmedo Cuaspud, Dary Mendoza, Gigliola Navarro, Juan Arias, Isabel Calle, Juliana Arcila-Galvis, Rafael Eduardo Arango Isaza

**Affiliations:** ^1^ Grupo de Investigación en Biotecnología Vegetal UNALMED - CIB, Universidad Nacional de Colombia, Facultad de Ciencias, Medellín, Colombia/Corporación para Investigaciones Biológicas, Medellín, Colombia; ^2^ Grupo de Investigación en Biotecnología Industrial, Universidad Nacional de Colombia, Facultad de Ciencias, Medellín, Colombia; ^3^ Grupo de Productos Naturales y Bioquímica de Macromoléculas, Universidad del Atlántico, Facultad de Ciencias, Barranquilla, Colombia; ^4^ Grupo de Investigación en síntesis orgánica, de polímeros y biotecnología aplicada (SINBIOTEC), Universidad EIA, Escuela de Ciencias de la Vida y Medicina, Envigado, Colombia; ^5^ Biosciences Institute, Newcastle University, Newcastle Upon Tyne, United Kingdom

**Keywords:** *Thevetia peruviana*, transcriptome, secondary metabolites, methyljasmonate, plant cells

## Abstract

*Thevetia peruviana* (Pers.) K. Schum is a tropical shrub with recognized ethnomedicinal applications associated with the presence of secondary metabolites (SMs), which exhibit cardiotonic, antioxidant, antimicrobial and anticancer activities. Previous studies have shown that methyl jasmonate (MeJA), when exogenously applied to *T. peruviana* cell cultures, activates the production of phenolic compounds (PCs), flavonoids (Fvs) and cardiac glycosides (CGs); however, the biochemical mechanisms involved in the MeJA-regulated biosynthetic pathways remain unknown. To deepen our understanding of the effect of MeJA on the secondary metabolism of *T. peruviana*, transcriptome sequencing was performed on suspension cell culture. A first draft transcriptome of *T. peruviana* was obtained, with an average N50 length of 3570 bp, comprising a total of 83126 unigenes. Differential gene expression analysis was conducted to evaluate the effects of treatment with 3 µM MeJA. In MeJA-treated cells, genes involved in the glycolytic pathway were upregulated, providing the necessary energy and metabolic precursors for SMs biosynthesis. Additionally, key genes in the biosynthesis of PCs (*HST, ALDH2C4*), Fvs (*SHT, FLS/F3H*, *FaGT6*) and CGs (ISPF, *TPS, SQS1, IPP2, CYP710A3, SCL14, DWF1*) were significantly upregulated in response to MeJA. Other notable effects of MeJA included the regulation of transcription factors (bHLH, MYB, bZIP, WRKY and ERF), which are involved in the biosynthesis of target metabolites. This *de novo* assembly of *T. peruviana* transcriptome provides a valuable resource for future research in functional genomics and metabolic engineering of bioactive SMs. Additionally, it offers new insights into the molecular mechanisms underlying the plant’s response to MeJA, paving the way for targeted strategies to enhance the production of pharmacologically relevant compounds.

## 1 Introduction

*Thevetia peruviana* (Pers.) K. Schum (family: Apocynaceae) is a plant native to Central America that grows in many tropical regions worldwide. In traditional medicine, it has been used to treat external wounds, infected skin, ringworm, tumors, and other conditions (Rahman et al., 2017). Additionally, the pharmacological potential of certain cardiac glycosides from *T. peruviana* (e.g., peruvoside and thevetins) has been explored for the treatment of congestive heart failure due to their positive inotropic effect, which is similar to that of digitalis drugs (Gozalpour et al., 2013). Recent studies also suggest that these cardiac glycosides may have therapeutic potential against various types of cancer, including leukemia, lung, breast, and liver cancers (Feng et al., 2016; Reddy et al., 2020; Kumavath et al., 2021). Moreover, other metabolites, such as flavonols, flavones, and flavanones, found in the leaves and fruits of *T. peruviana*, are recognized for their potent antioxidant (Rehman Khan et al., 2024), antibacterial (Arias et al., 2019), antifungal (Meena et al., 2021) and antiviral activities (Tewtrakul et al., 2002).

The biosynthetic pathways of secondary metabolites (SMs) in plants, including cardiac glycosides, terpenes, and certain flavonoids, are highly branched and complex. These processes occur, at least partially, in different cellular organelles, making their study particularly challenging (Ali, 2021). Additionally, plants are continuously exposed to various biotic and abiotic factors that can activate cellular signaling mechanisms, ultimately leading to the expression of genes encoding enzymes and proteins involved in specific biosynthetic pathways (Ncube et al., 2012). Jasmonic acid (JA) and methyl jasmonate (MeJA) are endogenous phytohormones that play a key role in the regulation of metabolic processes, reproduction, and defense against stress factors such as drought, high salinity, freezing, excessive exposure to UV radiation, heavy metal toxicity, micronutrient toxicity, pest attack, pathogen infection, among others (Ali and Baek, 2020). In the case of *T. peruviana*, suspension cell cultures were established with stable biomass production (ranging from 14 to 18 g dry weight per liter by day 14 of growth) (Arias et al., 2016; Mendoza et al., 2018). A subsequent study demonstrated that exogenous elicitation with MeJA significantly increases the accumulation of phenolic compounds (PCs), flavonoids (Fvs), and cardiac glycosides (CGs) (Mendoza et al., 2018; Mendoza, 2019; Cuaspud et al., 2025). However, the molecular mechanisms underlying JA/MeJA elicitation are not yet characterized in this species, which limits our understanding of their effects on processes such as: cell growth, regulation of defense gene expression, switching on and off of specific signaling pathways, redirection of carbon flux, biosynthesis of species-specific metabolites (e.g., cardiac glycosides and flavonoids), and other biological processes. Understanding these mechanisms is essential for identifying key genes or regulatory points in signaling pathways that are critical for the transformation of *T. peruviana* cell lines capable of overproducing SMs. In this context, transcriptomics, which involves the study of both mRNA and non-coding RNA, provides valuable insights into the transcriptome of a specific cellular state, reflecting gene expression dynamics.

Unlike the genome, the transcriptome is highly dynamic, varying across individuals, developmental stages, and environmental conditions (Wang et al., 2024). It serves as the essential first step, providing a reference for comprehensive studies aimed at understanding gene expression patterns, capturing dynamic changes across conditions, and identifying key genes and regulatory elements in SMs biosynthetic pathways (Younessi-Hamzekhanlu et al., 2022). Transcriptomic analyses have been successfully applied to study the expression of genes involved in the cardiac glycoside pathway in *Digitalis purpurea* plants. These studies have highlighted the upregulation of candidate genes for Scarecrow-like protein 14 (SCL14), delta^24^-sterol reductase (DWF1), and delta^8^–delta^7^-sterol isomerase HYDRA1 (HYD1), which promote cholesterol and cardiac glycoside biosynthesis in response to treatment with 100 µM of MeJA (Amiri et al., 2023). Transcriptome analysis has also been used to understand the transcriptional regulation mechanisms of MeJA-induced flavonoid biosynthesis in *Pyrus communis* callus cultures. This approach led to the identification of structural genes involved in the Fvs biosynthesis pathway, as well as the potential role of the MYB10-MYC2 molecular complex at the transcriptional level (Premathilake et al., 2020). Likewise, a transcriptomic analysis revealed differential expression of genes associated with the phenylpropanoid biosynthesis pathway, including transcription factors (TFs) such as MYC2, DELLA and, MYB111, in *Rosmarinus officinalis* cell suspension cultures treated with MeJA (50 and 100 µM), compared to the untreated control (Yao et al., 2021).

Currently, neither the genome nor the transcriptome of *T. peruviana* has been reported, nor have any studies been published on differentially expressed genes in this plant in response to MeJA. Here, we present for the first time a *de novo* transcriptome of *T. peruviana*, assembled from RNA-seq data obtained from leaves, roots and cell suspension cultures. A differential gene expression analysis was also carried out in the cell suspensions, treated and untreated with MeJA. This experimental approach demonstrated a relevant role for MeJA-responsive transcriptional regulators (e.g., TFs from the bHLH, MYB, bZIP, WRKY, and ERF families) in controlling the autoregulatory jasmonate cycle. Furthermore, it was shown that a MeJA-induced shift in oxidative status redirects the metabolism toward the production of antioxidant compounds, such as phenolics and flavonoids. The identification of a set of candidate defense genes associated with jasmonate signaling constitutes a valuable resource for genetic engineering studies of pharmaceutically valuable SMs in *T. peruviana* suspension cell cultures.

## 2 Materials and methods

### 2.1 Reagents

Folin-Ciocalteu reagent (2.0 N), methyl jasmonate (95%), quercetin (98%) and digoxin (97%) were purchased from Sigma-Aldrich Chemicals (St. Louis, MO). Gallic acid (≥ 98%), ursolic acid (≥ 90%), Molecular biology grade water and TBE 10X were purchased from Thermo Scientific.

### 2.2 Establishment of *T. peruviana* suspension cell culture

It was performed following the protocol previously developed by our research group and reported in Arias et al., 2010. Briefly, cell suspensions were established by inoculating 2–3 g of dry cells in 250 mL flasks with 100 mL of SH liquid sterile medium at room temperature (25°C) and 110 rpm.

### 2.3 Elicitation with MeJA

Initially, a 300 µM MeJA stock solution was prepared in EtOH:H_2_O (1:1; v/v) mixture and filter-sterilized using a 0.45 μm Millipore filter (Minisart^®^, Sartorius, Germany). *T. peruviana* plant cell cultures were treated with a 3 µM MeJA solution on day 6 of growth. The concentration and elicitation time were established in a previous study (Mendoza et al., 2018). To evaluate the influence of elicitation time, cells were harvested every 24 hours for six days (144 h). All experiments were performed in triplicate (biological replicates) using a destructive sampling method, in which the entire sample was processed at each harvest time. The experimental control consisted of a 50% (v/v) aqueous ethanol solution.

### 2.4 Extraction and quantification of SMs

The harvested cells (biomass) at different culture times were freeze-dried at 5°C and 1 Pa for 52 h using a Syclone 18N lyophilizer, then pulverized with a mortar and pestle. Subsequently, 0.3 g of powder was mixed with 15 ml of an 80% (v/v) aqueous ethanol solution and subjected to ultrasonic extraction (40 Hz) at 30°C for 30 min. The extracts were then centrifuged at 3000 rpm for 10 minutes, and the supernatant was collected (Mendoza et al., 2018). SMs such as TPC, TFv, TCG, and TT were measured using colorimetric techniques (**Supplementary Material: Materials and Methods**).

### 2.5 Total RNA extraction, cDNA library construction, and sequencing

MeJA-treated and untreated suspension cells were harvested at the first time point where the difference in secondary metabolite accumulation was positive and significant (p-value <0.05), then the samples were snap-frozen in liquid nitrogen until further use. RNA was isolated from tissue samples using the CTAB method (Rajakani et al., 2013). The quantity and quality of RNA samples were assessed using a Nanodrop spectrophotometer (Thermo Fisher Scientific) and agarose gel electrophoresis. For qPCR analysis, DNA digestion was carried out using DNase I (Thermo Fisher Scientific), followed by conventional PCR to confirm complete degradation of DNA. cDNA synthesis was performed using the Maxima H Minus First Strand cDNA Synthesis Kit (with dsDNase) (Thermo Scientific). The library preparation and sequencing of RNA of plant cell suspensions, both treated and untreated with MeJA (in triplicate), were done by Beijing Genomics Institute – BGI (Shenzhen, Guangdong, China). The RNA library was enriched using Oligo dT selection, and sequencing was performed with MGI DNBseq™ technology (Fehlmann et al., 2016). In addition, a leaf sample and a root sample were included to monitor the RNA extraction process.

### 2.6 Differential expression analysis and identification of genes related to secondary metabolite production

For more details on the transcriptome assembly and annotation, including the parameters used to assess data quality and assembly performance, please refer to the **Supplementary Table S1**.

To estimate gene expression levels, reads from three without treatment samples (Ctrl) and three MeJA-treated samples were aligned to *de novo* assembled transcriptome using the RSEM software (Li and Dewey, 2011), generating expected count values for each transcript. These values were compiled into a count matrix. All downstream analyses were conducted in the R statistical environment using the Bioconductor platform. The edgeR (Robinson et al., 2010) and limma (Ritchie et al., 2015) packages were used for data processing and statistical analysis. To correct for differences in sequencing depth, raw counts were transformed into counts per million (CPM). Normalization was performed using the trimmed mean of M-values (TMM) method (Robinson and Oshlack, 2010), which adjusts for compositional biases among libraries.

Differential expression analysis was conducted between MeJa-treated and Control samples using the limma-voom workflow. Statistical significance was defined as a *p*-value < 0.05 and an absolute fold change (FC) greater than 1.5.

The metabolic pathways involved in the biosynthesis of SMs known to be produced by *T. peruviana* as well as their regulatory mechanisms were inferred using databases containing information from other plants, such as KEGG (Kanehisa et al., 2007) and Cathacyc (Van Moerkercke et al., 2013). Gene sequences associated with these pathways were retrieved from the *T. peruviana* transcriptome by sequence similarity. Differentially expressed genes were identified, counted, and classified according to their respective metabolic pathways or their roles in regulation (e.g., transcription factors). Finally, these genes were categorized into one of two groups: upregulated or downregulated. The statistically significant enrichment of specific Gene Ontology (GO) terms was analyzed using GO enrichment of the BiNGO plugin of Cytoscape v3.10.2 (Maere et al., 2005). And VANTED was used for the graphical representation of the pathways (Rohn et al., 2012) using the KEGG pathways database of *Arabidopsis thaliana.*

### 2.7 Real-time qPCR analysis

To validate the RNA-seq results, RT-qPCR analyses were performed. Gene-specific primers were designed using Primer3Plus (Untergasser et al., 2007), using transcript sequences from the assembly, and confirmed using BLASTn against the *T. peruviana* transcriptome generated in this study. The sequences of all primers used are provided in **Supplementary Table S2**. RT-qPCR was conducted using the Maxima SYBR Green/ROX qPCR Master Mix (2X) from Thermo Scientific in 96-well optical PCR plates on the AriaMX Real-Time PCR System (Agilent Technologies). Transcript levels of each gene in the different tissue samples were normalized 177 to the transcript levels of and an internal control gens of family *SAND*; vacuolar fusion protein 178 *MON1* (Pollier et al., 2014; Sheshadri et al., 2018). For each tissue sample, at least three independent biological replicates were analyzed, with three technical replicates for each biological replicate.

### 2.8 Statistical analysis

The data collected was analyzed using the free statistical software R. The quantification of TPC, TFv, TCG, and TT was performed in triplicate and expressed as mean values with their corresponding standard deviations (SD). Assessments of treatment variations were conducted through one-way analysis of variance (ANOVA) at a significance level of 0.05, prior confirmation of the assumptions of homogeneity of variance, normal distribution of residuals and non-additivity. Multiple pairwise comparisons were then performed using Tukey’s honest significant difference test (Tukey’s HSD), with outcomes reported as 95% confidence intervals (95% CI).

## 3 Results

### 3.1 Effect of MeJA treatment on *T. peruviana* SMs production

To identify the optimal post-elicitation timepoint with 3.0 µM MeJA, the production of SMs (TPC, TFv, TCG, and TT) was assessed following methods described in section 2.4.

**Figure 1:**
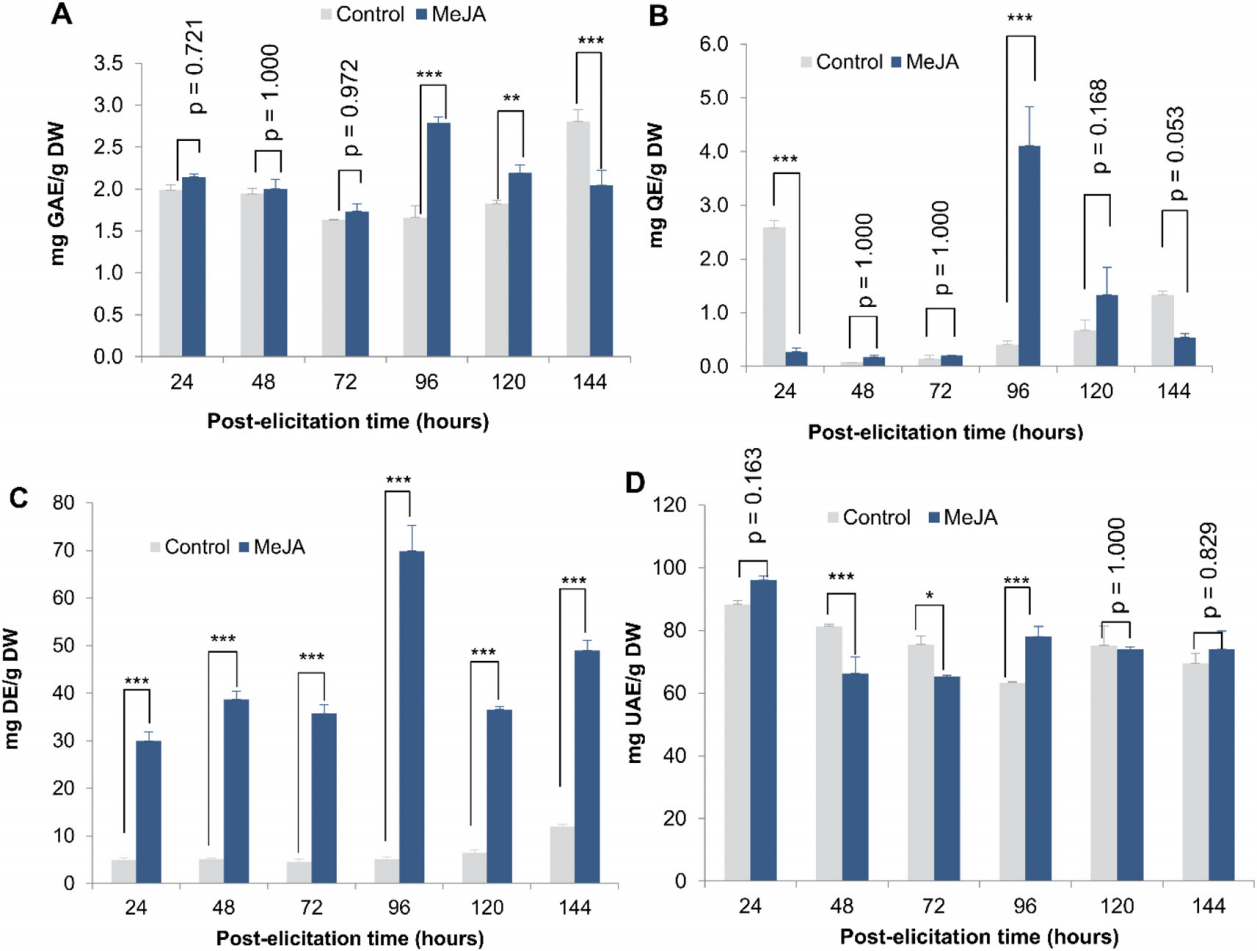
Effect of elicitation time with MeJA (3 µM) on secondary metabolites production. Total phenolic compounds **(A)**, Total flavonoids **(B)**, Total cardiac glycoside **(C)**, and Total triterpenoids **(D)** production in cell suspension cultures of *T. peruviana* elicited with 3 µM of MeJA. Statistical significance levels are indicated as follows: ***p < 0.001, **p < 0.01 and *p < 0.05.

No significant difference in TPC content was observed between 24 and 72 h post-elicitation. However, at 96 h post-elicitation (mean difference from control = -1.129; p-value < 0.0001; LCL = -1.422 and UCL = -0.836) and 120 h post-elicitation (mean difference from control = -0.367; p-value = 0.007; LCL = -0.660 and UCL = -0.074), TPC concentrations were 1.68-fold and 1.2-fold higher, respectively, in elicited cultures compared to the control (**Figure 1A**). A similar trend was observed for Fvs quantification, with the highest concentration recorded at 96 h post-elicitation (4.107 ± 0.727 mg QE/gDW) (mean difference from control = -3.700; p-value < 0.000; LCL = -4.499 and UCL = -2.902) (**Figure 1B**).

In contrast, MeJA induced an increase in TCG production as early as 24 h post-elicitation, with the highest concentration observed at 96 h (69.877 ± 5.300 mg DE/g DW) post-elicitation (**Figure 1C**). Additionally, TT concentration significantly increased in MeJA-treated cultures at 96 h post-elicitation (78.085 ± 3.184 mg UAE/g DW) (mean difference from control = -14.766; p-value = 0.0004; LCL = -24.094 and UCL = -5.437), after which no significant difference was observed compared to the control without elicitor (**Figure 1D**).

### 3.2 Transcriptomic insights into MeJA-Induced gene expression in *T. peruviana*

To investigate the effect of MeJA on genes involved in the biosynthetic pathways of PCs, Fvs, Tpds and CGs, transcriptome sequencing was performed on *T. peruviana* suspension cells harvested 96 hours post-MeJA elicitation, the time point at which the highest concentration of PCs, Fvs and CGs were achieved. Since no reference genome or transcriptome for *T. peruviana* is available in public databases, we generated and annotated a *de novo* transcriptome to enable gene expression quantification.

**Figure 2:**
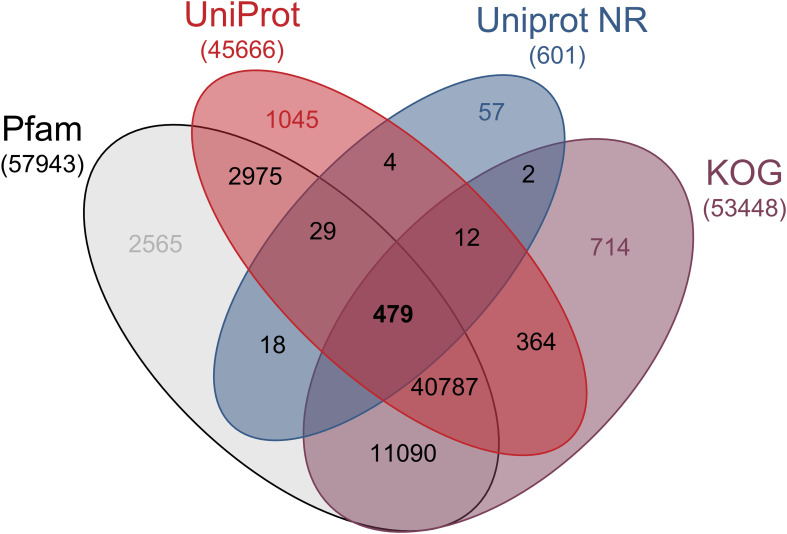
Venn diagram with gene annotation in protein sequence databases. Venn diagram showing the number of differentially expressed transcripts homologous to known annotated genes from PFAM, UPROT, NCBI-NR y KOG protein databases.

A total of 520 million high-quality reads were obtained, with an average Phred score >30 (**Supplementary Table S1**). *De novo* assembly of these reads resulted in a draft transcriptome comprising 83126 putative genes. Open reading frames (ORFs) were predicted from the assembled transcripts and compared against the Clusters of Eukaryotic Orthologous Groups (KOG) database, achieving 98.0% coverage of eukaryotic lineage genes. Among the 83126 sequences, 621 (0.7%), 45695 (54.9%), 57963 (69.7%), 53468 (64.3%) unigenes showed significant similarity to known proteins in NR, Uniprot, Pfam, and KOG database, respectively (**Figure 2**). The results of BLASTp against different databases, along with their functional annotations, are summarized in **Supplementary Table S4**.

**Figure 3:**
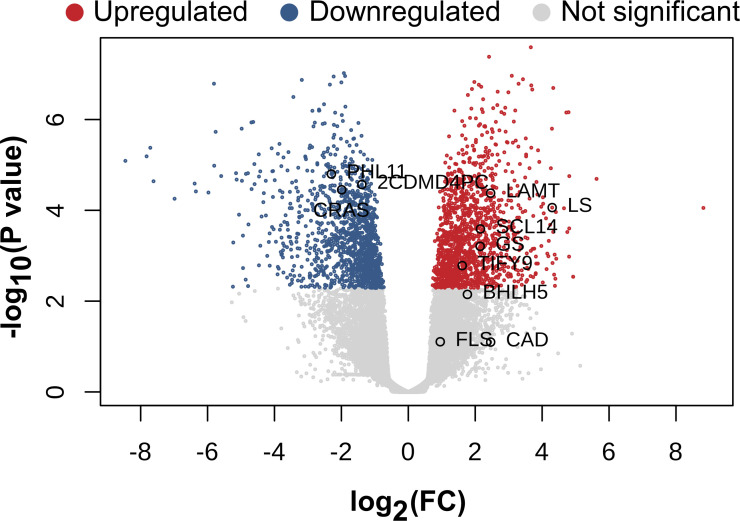
Genes with differential expressions. Volcano plot of differential gene expression analysis between the control samples and the samples treated with MeJA.

Considering only a p-value < 0.05, 4972 genes were upregulated and 5314 were downregulated while 15060 genes did not exhibit significant changes Additionally, when a stricter filtering was applied (p-values < 0.05, FC > 1.5) with p-values adjusted < 0.05, 407 upregulated genes, 356 downregulated genes and 24583 non-significant genes were identified (**Figure 3**).

Among the top 10 upregulated genes, UDP glycosyltransferase-9 (UDP9), Trichome differentiation protein (GL1), and transcription factor BHLH18 exhibited fold changes of 27.66, 26.99, and 25, respectively. In contrast, Indole-3-acetic acid amino synthetase, polygalacturonase, and auxin efflux carrier components were among the most downregulated genes with fold changes of 352.1, 225.97, and 127.11, respectively (**Supplementary Table S5**).

**Figure 4:**
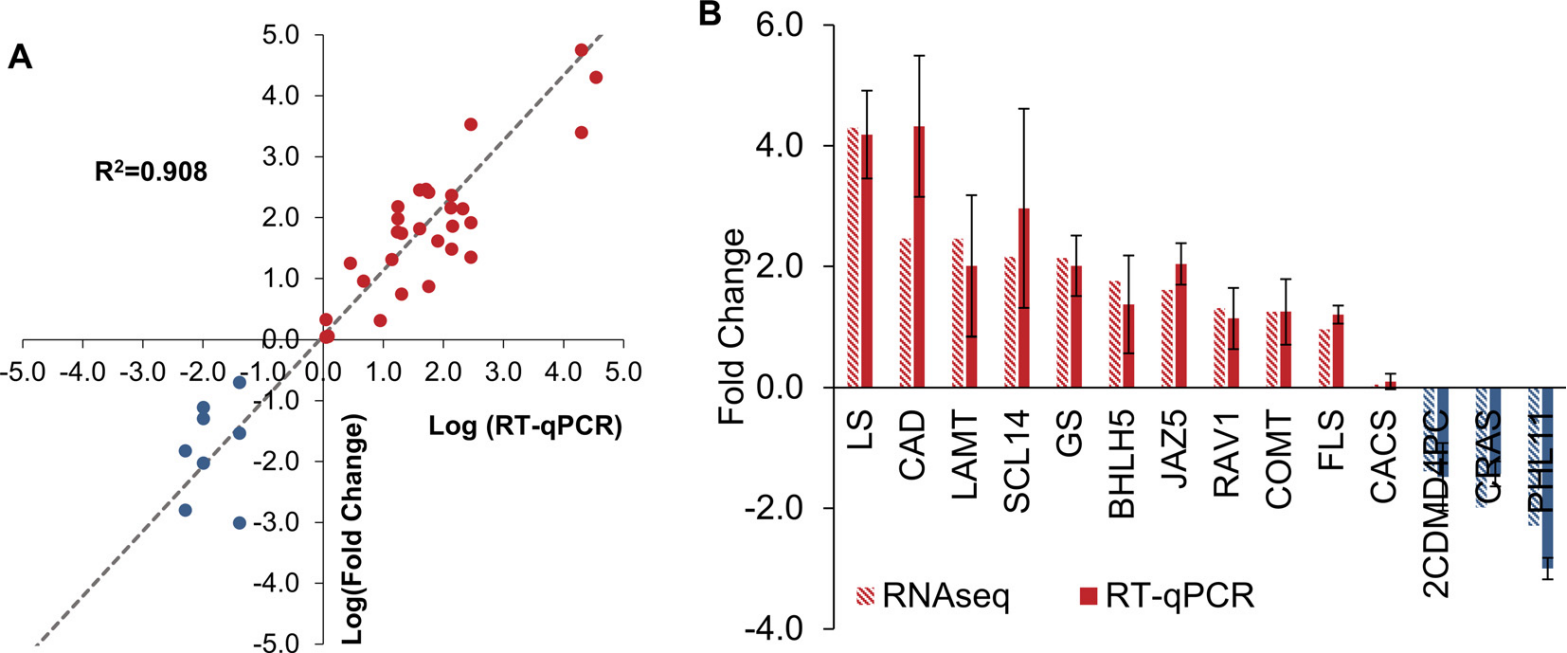
Validation of transcriptome. Correlation between qRT-PCR and RNA-seq for 14 secondary metabolite-related genes **(A)**. The relative expression of genes from RNA-seq by qRT–PCR (DEGs) **(B)**.

To validate the *T. peruviana* transcriptome, representative differentially expressed genes associated with the secondary metabolism and its regulation were selected. These included key enzymes from the phenylpropanoid and flavonoid biosynthetic pathways: Caffeoyl-CoA O-methyltransferase 1 (*COMT*), Cinnamyl-alcohol dehydrogenase (*CAD*), and Flavonol synthase (FLS), as well as genes from the MEP pathway and terpenoid biosynthesis: 2-C-methyl-D-erythritol 4-phosphate cytidylyltransferase (*2CDMD4PC*), Geraniol synthase (GS), Lupeol synthase (*LS*), α-amyrin synthase (*CRAS*), and Longanic acid O-methyltransferase (*LAMT*). The Scarecrow-like protein 14 (*SCL14*) gene implicated in the regulation of cardiac glycosides—characteristic metabolites of this species—was also considered. Additionally, transcription factors involved in the regulation of specialized metabolites were included, such as basic helix-loop-helix transcription factor 5 (BHLH5), MYB-like transcription factor PHL11 (PHL11), and ABI3/VP1-related transcription factor 1 (RAV1), along with Jasmonate ZIM-domain protein 5*(JAZ5)*, a repressor linked to jasmonate-mediated signaling. The clathrin adaptor complex subunit (CACS) and SAND family protein genes, previously reported as stable in transcriptomic studies focused on flavonoid pathways were used as expression controls to support the assessment of assembly quality and transcriptional consistency (Pollier et al., 2014; Sheshadri et al., 2018). The qRT-PCR results showed expression patterns consistent with those obtained from RNA-seq analysis with an average R²= 0.909 (**Figure 4**). These findings confirm the reliability and accuracy of the gene expression data derived from RNA-seq.

### 3.3 Identification of dysregulated pathways, including SM biosynthesis pathways in *T. peruviana* upon MeJA treatment

**Figure 5:**
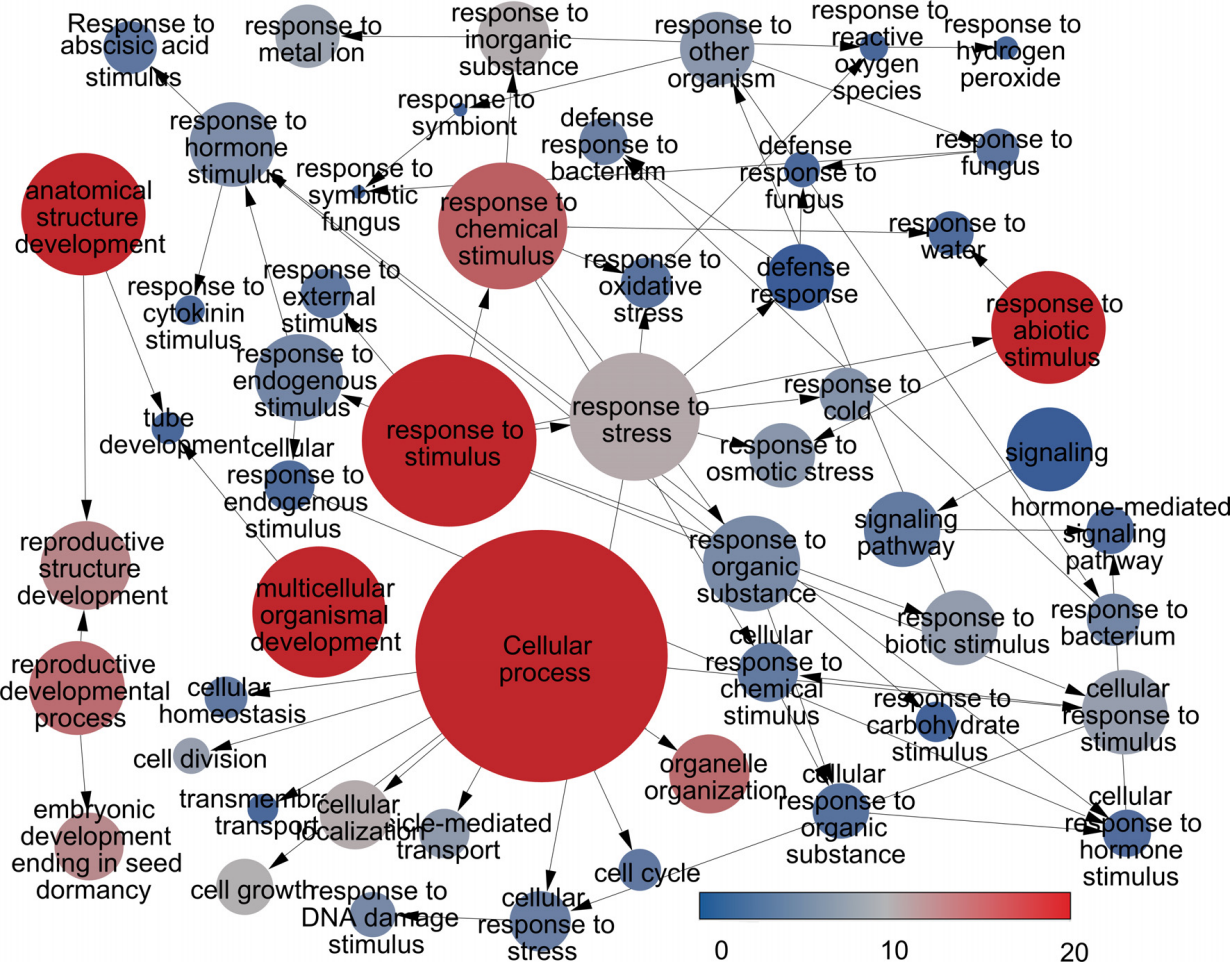
Significant enriched Gene Ontology (GO) terms are depicted with a color scale representing the significant level of enrichment. The size of each sphere corresponds to the number of annotated genes associated with each GO term and the color indicating the significance.

This analysis facilitated the assignment of GO terms to the complete set of differentially expressed genes (DEGs). In the general category of biological processes, terms such as ‘cellular process (GO:0009987)’, ‘metabolic process (GO:0008152)’, ‘primary metabolic process (GO:0044238)’, ‘response to stimulus (GO:0050896)’, ‘biological regulation (GO:0065007)’, ‘response to chemical stimulus (GO:0042221)’, and response to stress (GO:0006950) emerged as the most significantly enriched GO terms. On the other hand, in the cellular component category, prevalent GO terms among the DEGs included ‘localization (GO:0051179)’ and ‘anatomical structure development (GO:0048856)’. As for molecular functions, the most prevalent terms were ‘signaling pathway (GO:0023033)’ (**Figure 5**). **Supplementary Figure S3** presents the GO terms with differential expression, categorized into three levels of expression.

Significant enrichment was revealed in several pathways. The top ten pathways identified include Metabolic pathways (132 genes), Biosynthesis of secondary metabolites (77 genes), Plant hormone signal transduction (28 genes), Starch and sucrose metabolism (21 genes), Plant-pathogen interaction (13 genes), Biosynthesis of cofactors (12 genes), Glycolysis/Gluconeogenesis (12 genes), Phenylpropanoid biosynthesis (11 genes), ABC transporters (11 genes), and Pentose and glucuronate interconversions (11 genes). The complete list of genes is shown in **Supplementary Table S3**.

### 3.4 Identification of differentially expressed genes associated with SMs

**Figure 6:**
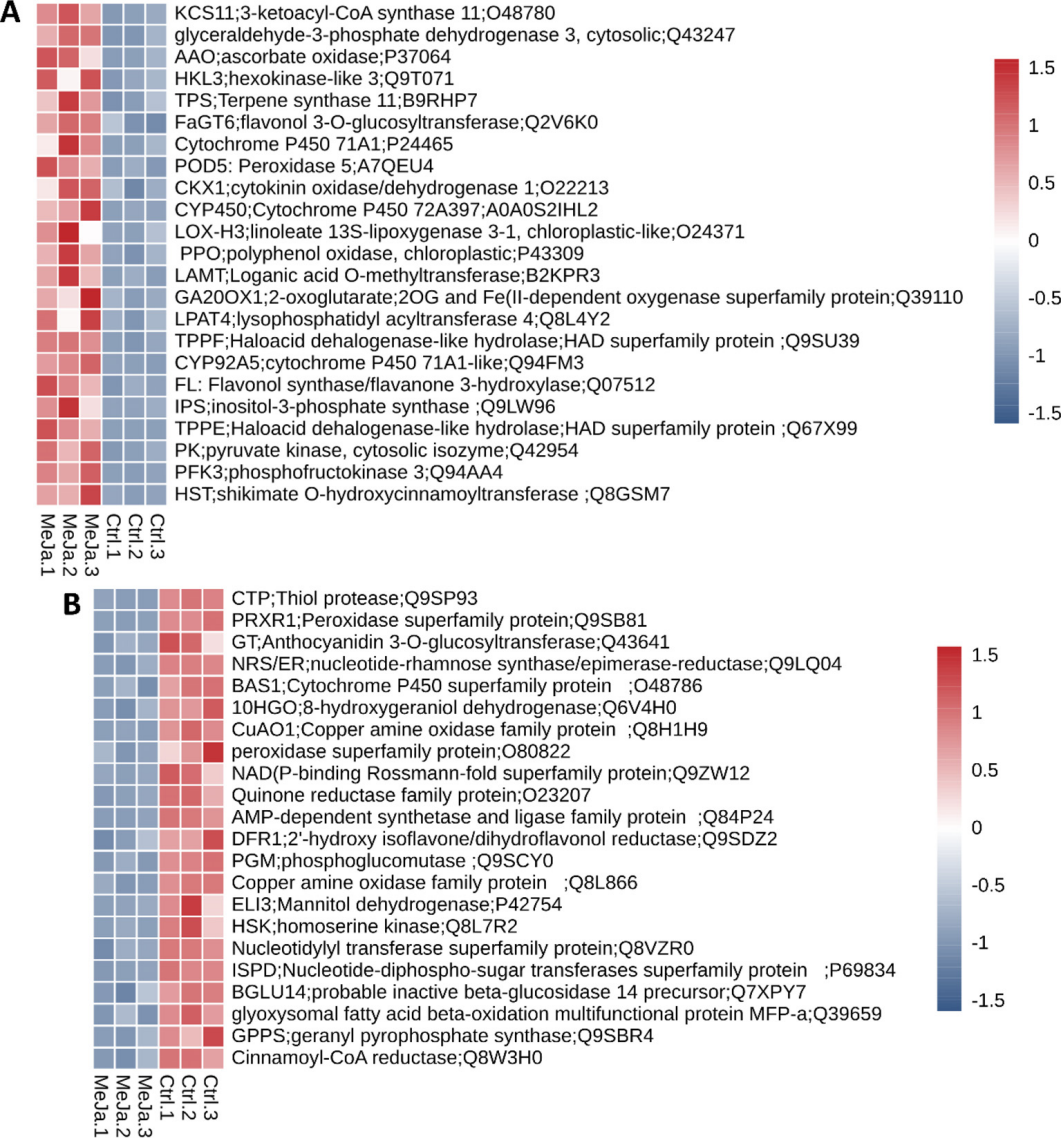
DEGs associated with secondary metabolism. Heatmap plots highlight both upregulated **(A)** and downregulated genes **(B)**. The color scale, ranging from -6 (dark blue) to 6 (bright red) represents the z-score normalized expression values observed across the three biological samples.

The analysis of genes associated with secondary metabolism based on the KEGG database, revealed that 55 genes exhibit upregulation (LogFC > 0.58), while 44 are downregulated (LogFC < -0.58). Among the most significantly upregulated genes, notable examples include *HST* (LogFC = 3.91), a shikimate O-hydroxycinnamoyltransferase involved in the biosynthesis of phenolic compounds; *PFK3* (LogFC = 3.51), phosphofructokinase 3, which provides energy and metabolic precursors for the synthesis of secondary metabolites; *PK* (LogFC = 3.50), pyruvate kinase, suggesting an increased energy demand; *TPPE* (LogFC = 3.26), a haloacid dehalogenase hydrolase; and *IPS* (LogFC = 3.15), inositol-3-phosphate synthase, a key enzyme in cell signaling pathways critical for adaptation and stress response. This upregulation profile suggests a metabolic shift towards the active synthesis of specialized compounds such as phenols, flavonoids, and other SMs, enhancing the plant’s defensive capabilities (**Figure 6A** and **Supplementary Table S6**).

In contrast, the downregulated genes appeared to be associated with processes that, although important, are inactivated in favor of more urgent pathways under MeJA treatment. Examples include *CTP* (LogFC = -6.35), a thiol protease involved in protein degradation; *PRXR1* (LogFC = -3.18), a peroxidase, indicating a potential reorganization of the plant’s antioxidant strategies; GT (LogFC = -2.78), an anthocyanidin 3-O-glucosyltransferase involved in flavonoid biosynthesis whose function may be temporarily deprioritized in favor of more critical pathways; *NRS/ER* (LogFC = -2.46), an enzyme involved in nucleotide-rhamnose synthesis and the formation of secondary sugars; and *BAS1* (LogFC = -2.44), a *CYP450* family member, which is key in SMs biotransformation and whose repression suggests an adjustment in metabolic flux (**Figure 6B**; **Supplementary Table S6**).

**Figure 7:**
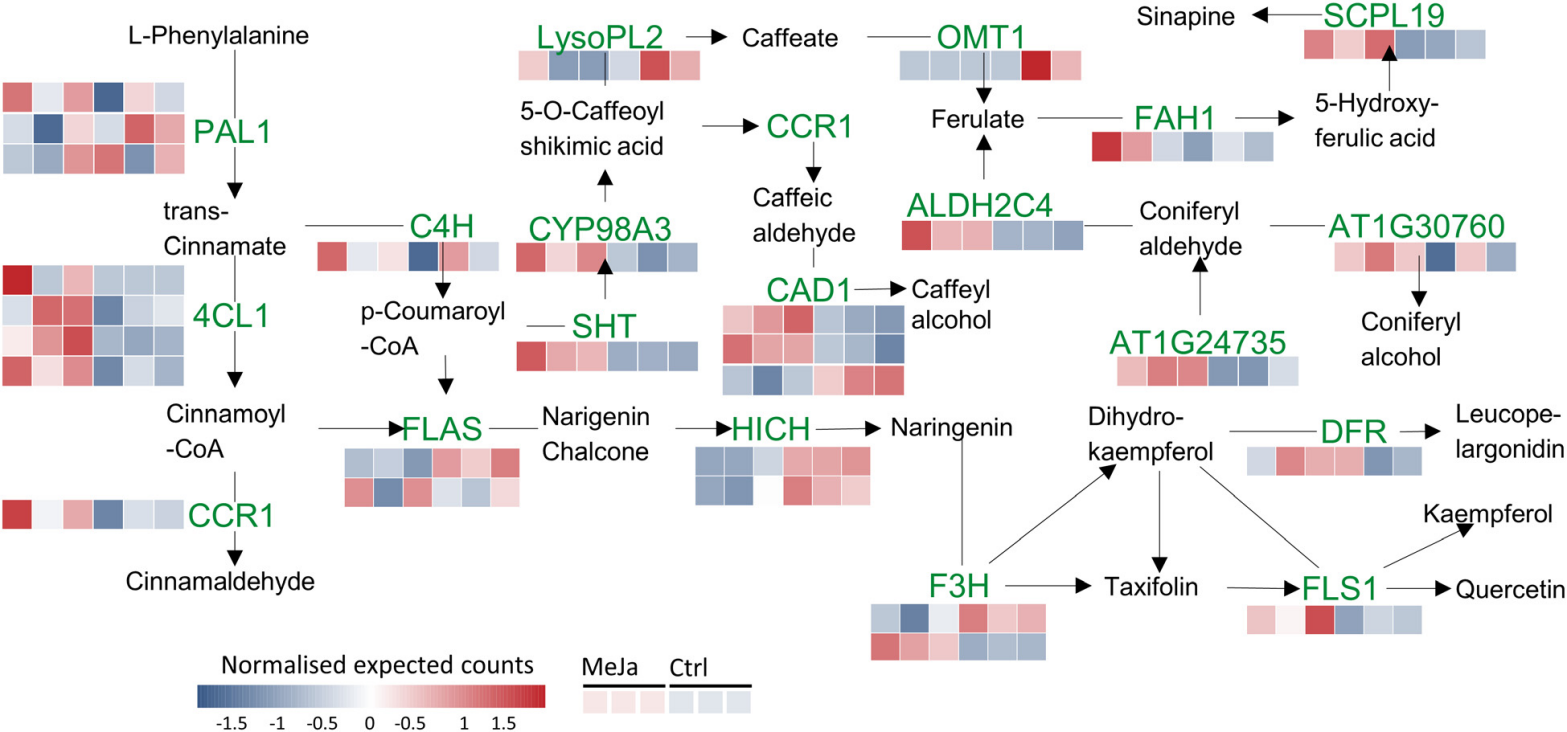
Phenylpropanoid and flavonoid biosynthesis pathways. Red and blue squares indicate transcripts that are upregulated and downregulated, respectively, in *T. peruviana* cell cultures due to treatment with 3 µM MeJA.

MeJA serves as a potent inducer of secondary metabolites by modulating the expression of ZIM-domain genes such as *JAZ5*(LogFC=1.61)*, JAZ6* (LogFC=1.31)*, JAZ8* (LogFC=2.21) and *OPDA-OPR3* (LogFC=2.17), whose degradation activates key transcription factors (ERF, WRKY, bHLH, and MYB) (**Figure 7A**). Among the most enriched metabolic pathways was the phenylpropanoid pathway with 13 DEGs identified between the control and treatment samples, including 4-Coumarate ligase I (*4CL1*), cinnamoyl-CoA reductase 1 (*CCR1*), shikimate hydroxycinnamoyl transferase (*SHT*), flavonol synthase/flavanone 3-hydroxylase (*FLS/F3H*), cinnamyl alcohol dehydrogenase (*CAD*), chalcone-flavanone isomerase (*HICH*), and aldehyde dehydrogenase (*ALDH2C4*). These enzymes are involved at the start of the phenylpropanoid pathway and are key enzymes in the synthesis of methyl erythritol phosphate (MEP) derivatives (**Figure 7B**). For genes such as *PAL1*, *4CL1*, and *CAD1*, the presence of more than one colored band suggests the detection of multiple isoforms. The information and names of the genes with differential expression in this biosynthetic pathway can be seen in **Supplementary Table S4**.

**Figure 8:**
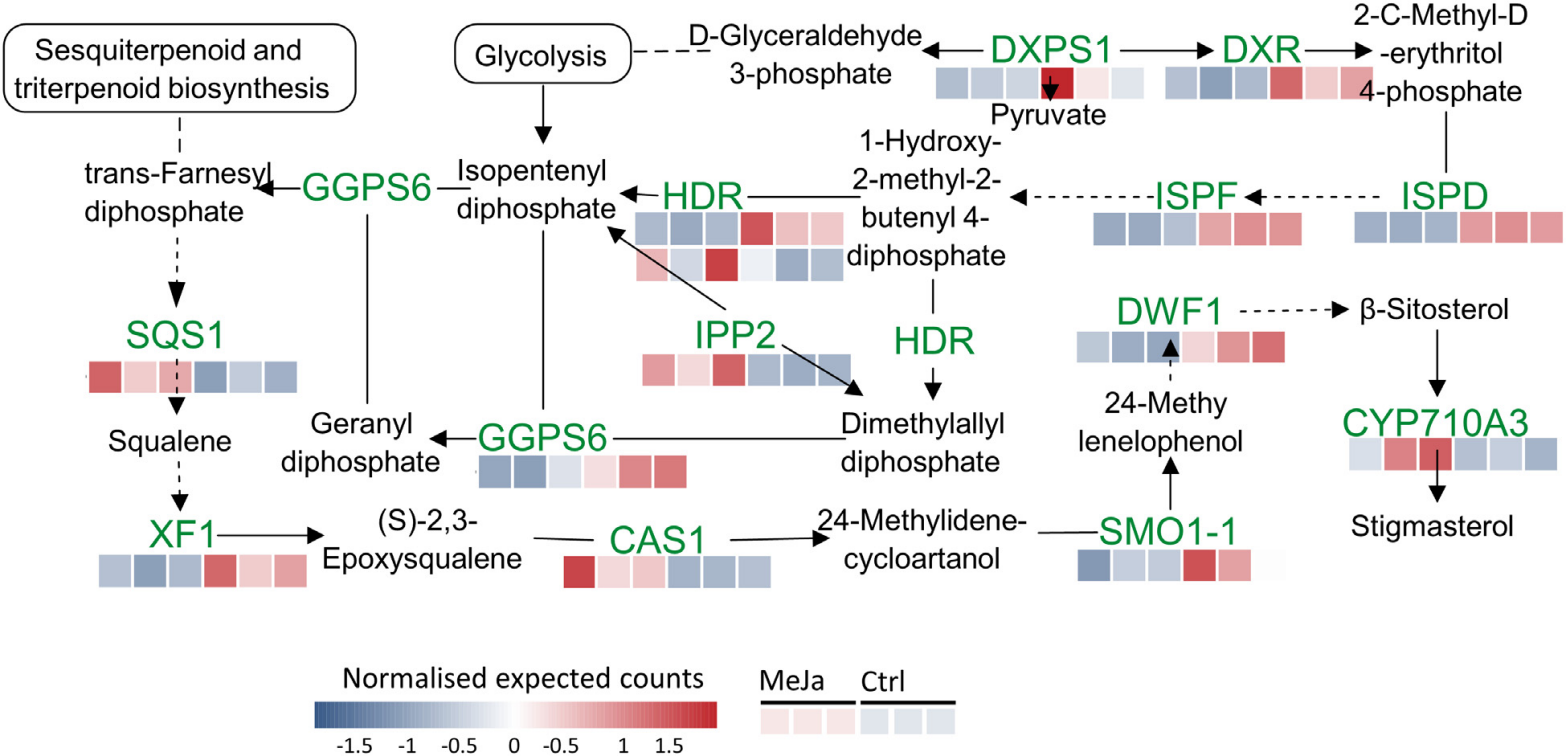
Terpenoid backbone and steroid biosynthesis pathway. Red and blue squares indicate transcripts that are upregulated and downregulated, respectively, in *T. peruviana* cell cultures due to treatment with 3 µM MeJA.

Another enriched metabolic pathway is the terpenoid backbone biosynthesis, in which five genes and nine DEGs were identified. These include delta(24)-sterol reductase (*DFW1*), 1-deoxy-D-xylulose 5-phosphate reductoisomerase (*DXR*), geranylgeranyl pyrophosphate synthase (*6GGPS6*), 4-hydroxy-3-methylbut-2-enyl diphosphate reductase (*HDR*), isopentenyl-diphosphate delta-isomerase II (*IPP2*), 2-C-methyl-D-erythritol 4-phosphate cytidylyltransferase (ISPD), 2-C-methyl-D-erythritol 2,4-cyclodiphosphate synthase (*ISPF*), squalene synthase 1 (*SQS1*) and squalene epoxidase 1 (*XF1*) (**Figure 8**). Details and gene names with differential expressions in this biosynthetic pathway are provided in **Supplementary Table S4**.

### 3.5 Identification of TFs

**Figure 9:**
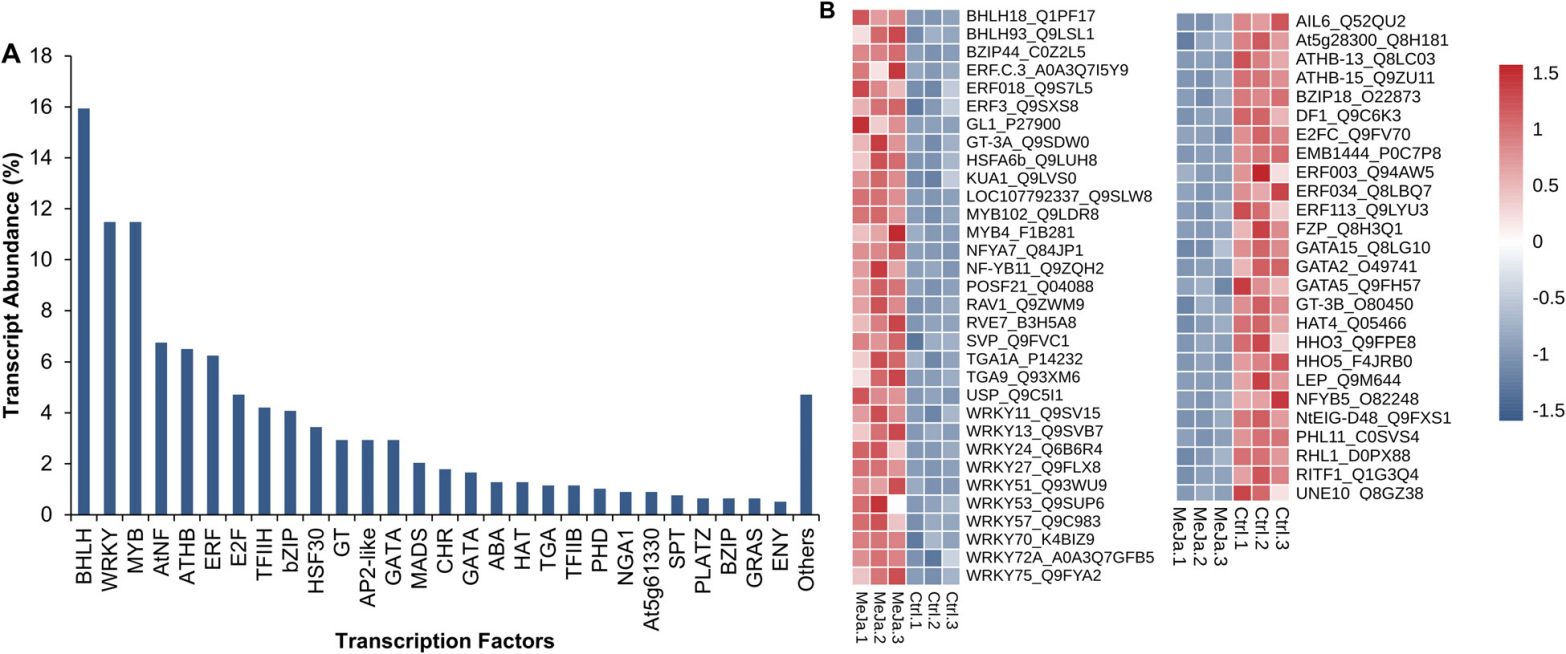
Transcription Factors. These factors were identified using the TFs database. Transcripts were counted as isoforms **(A)**. Differentially expressed transcription factors are shown **(B)**.

The distribution of transcription factors reveals that BHLH is the most abundant, accounting for 15.94% of the total, highlighting its central role in regulating key processes such as secondary metabolite biosynthesis, development, and stress responses. WRKY and MYB, both with an abundance of 11.48%, further support this trend, being widely associated with the activation of genes involved in defense and adaptation. At intermediate levels, E2F (4.72%), TFIIH (4.21%), and bZIP (4.08%) are likely to play secondary, yet crucial roles in regulating specific pathways. Similarly, factors like HSF30 (3.44%), AP2-like, GT, and GATA (each at 2.93%) are involved in stress-specific responses or the control of distinct metabolic pathways. Finally, less abundant factors such as PLATZ, GRAS, and ENY (0.64–0.51%) suggest a highly specialized regulatory role, potentially restricted to specific conditions (**Figure 9A**).

From the total of 784 transcription factors (TFs) identified, 58 exhibited differential expression between untreated *T. peruviana* samples and those treated with *MeJA*. Transcription factors from the bHLH and WRKY families stood out significantly. For instance, bHLH18 displayed strong upregulation with a LogFC of 4.65, suggesting its potential role in regulating critical pathways, such as stress response. Similarly, WRKY53 (LogFC = 3.28), WRKY13 (LogFC = 3.07), HSFA6b (LogFC = 2.49), and MYB102 (LogFC = 2.05) were among the most upregulated, reinforcing their established role in responding to biotic and abiotic stimuli. In contrast, genes like ERF003 (LogFC = -2.76) and ERF034 (LogFC = -3.28) exhibited significant downregulation, potentially indicating a repressive role in specific hormonal signaling pathways or environmental adaptation. Additionally, genes from the GATA family, such as GATA2 (LogFC = -3.35), were also downregulated, which may reflect a reduced contribution under the experimental conditions. Overall, the results suggest that the WRKY family predominantly regulates gene expression, with multiple upregulated members likely prioritizing the activation of defense responses. Meanwhile, the ERF family appears to play a dual role, potentially downregulating secondary metabolic processes to prioritize other critical pathways (**Figure 9B**; **Supplementary Table S7**).

**Figure 10:**
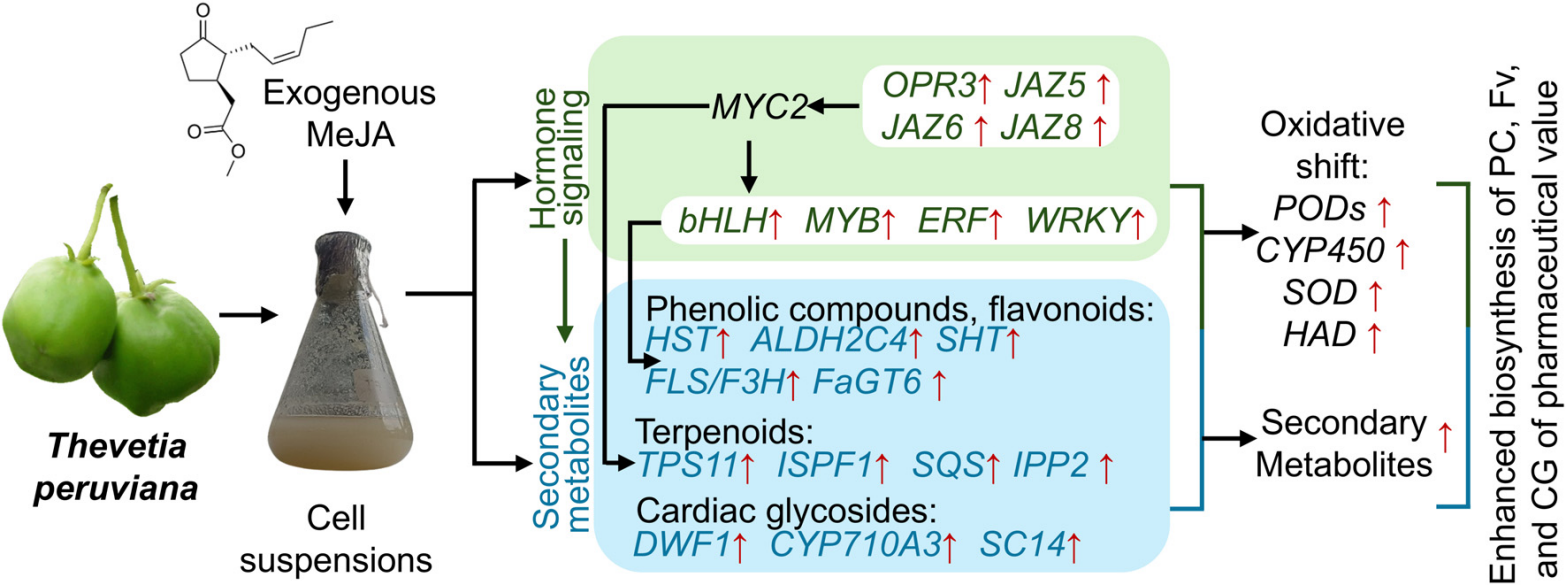
Working model of the effect of MeJA on gene expression in cell suspensions of *Thevetia peruviana*.

This study in *Thevetia peruviana* revealed that MeJA induces the expression of JAZ-domain proteins (*JAZ5, JAZ6*, and *JAZ8*), which in turn modulate the activity of key transcription factors such as bHLH, MYB, WRKY, and bZIP. These transcription factors orchestrate the expression of structural genes involved in the biosynthetic pathways of phenolic compounds (e.g., *HST*, *ALDH2C4*), flavonoids (e.g., *FLS*), triterpenoids (e.g., *TPS11*, *SQS*), and cardiac glycosides (e.g., *DWDF1*, *SCL14*), leading to increased production of secondary metabolites. Additionally, MeJA treatment induces an oxidative shift characterized by the upregulation of antioxidant-related genes, including *PODs*, *SOD*, and *HAD*. Collectively, this model offers a comprehensive overview of the molecular mechanisms by which MeJA promotes the biosynthesis of pharmacologically relevant secondary metabolites, reinforcing the biotechnological potential of *T. peruviana* in plant cell culture systems (**Figure 10**).

## 4 Discussion

### 4.1 MeJA increases the accumulation of TPC, TFv and TCG in *T. peruviana*

Exogenous application of MeJA, either alone or in combination with other elicitors, has been widely used to induce the biosynthesis and accumulation of specialized SMs (such as indole alkaloids) in both tissue and suspension cell cultures of plants of the Apocynaceae family (Akhgari et al., 2019; Ibrahim et al., 2021). Here, we demonstrate that MeJA (3 µM) induces the biosynthesis of TPC and TFv at 96 h post-elicitation, whereas TCG biosynthesis is activated as early as 24 h post-elicitation, reaching its maximum production at 96 h. These results indicate that the metabolic effects of MeJA in *T. peruviana* cell cultures are clearly time-dependent (**Figure 1**). Similar findings were reported in a previous study, where MeJA treatment significantly increased the content of TPC (1.49-fold) and TFv (2.55-fold) levels compared to the control (Mendoza et al., 2018).

Regarding cardiac glycosides, although their biosynthetic pathway is not yet fully understood, it is known that triterpenes and CGs share common metabolic precursors, such as 2,3-epoxide squalene (Thimmappa et al., 2014; Pandey et al., 2016). Consequently, the rapid activation of the CGs anabolic pathway likely depletes the precursors required for triterpenoid biosynthesis, providing a plausible explanation for the less pronounced effect of MeJA on terpenes production compared to CGs.

### 4.2 *De-novo* transcriptome of *T. peruviana* revealed large functional unigenes

Although metabolite quantification methods offer valuable insights into the effects of MeJA on *T. peruviana* cell cultures, they provide limited information about the underlying biochemical mechanisms of MeJA signaling. Transcriptome studies based on the Illumina platform have been successfully used to identify genes involved in the synthesis of specialized metabolites *in vitro* cultures of MeJA-treated medicinal plant cells, such as *C. roseus* (Pan et al., 2018) and *Rubia yunnanensis* (Zhang et al., 2022).

Illumina sequencing of *T. peruviana* generated approximately 540 million reads. Most reads for transcriptome assembly (74%) were obtained from suspension-cultured cell samples, while root and leaf tissues contributed the remaining 26%. Importantly, these latter samples were included exclusively for transcriptome assembly purposes and were not used in subsequent analyses. Optimization of the *de novo* assembly process resulted in approximately 194233 unique transcripts. Of these, 65% -corresponding to 83,126 unigenes- showed homology with sequences available in public repositories, while the remaining 35% were considered specific to *T. peruviana* (**Figures 2**, **3**).

Gene ontology (GO) and KEGG pathway analyses revealed a high activation of phenylpropanoid biosynthesis and carbohydrate metabolism in *T. peruviana*. Additionally, genes associated with plant hormone signal transduction and plant-pathogen interaction were highly represented. Although *de novo* reconstruction of a complete transcriptome using the Illumina RNA-seq method has inherent limitations, mainly due to challenges in contig assembly that hinder the full recovery of homologous transcripts (Kang et al., 2024). The identification of many unigenes related to metabolic pathways (132 genes) and cellular processes (160 genes) in *T. peruviana* allowed the identification of transcripts involved in the regulation of MeJA-dependent signaling pathways, which in turn activate specialized pathways for the biosynthesis of secondary metabolites of pharmaceutical value in *T. peruviana*. (**Figure 4**).

### 4.3 Exogenous-MeJA regulates both JA biosynthesis/JA signaling in *T. peruviana*

Recently, the study of the effect of exogenous MeJA on the biosynthesis of specialized SMs and cell growth has generated considerable interest in the field of plant biotechnology, particularly in *in vitro* cell and tissue cultures. JAs are oxylipins (oxygenated derivatives of fatty acids) synthesized by the octadecanoid/hexadecanoid pathways. In our study, transcriptomic analysis revealed that treatment with low concentrations of MeJA (3 µM) induces the expression of endogenous JA biosynthesis genes. Among the genes upregulated by MeJA are LOX-H3 and 12-oxo-phytodienoate reductase 3 (OPDA-OPR3). LOX-H3 is a lipoxygenase which promotes *de novo* synthesis of oxylipins from lipids containing a cis, cis-1,4-pentadienolinolenic acid structure. Linolenic acid (LA; 18:3), a polyunsaturated lipid abundant in plant cell membranes, is the preferred substrate of LOX-H3, which oxidizes it to 13-hydroperoxylinoleate (13-HPODE), a key oxylipin precursor in JA biosynthetic pathway (Creelman and Mulpuri, 2002). 13-HPODE is subsequently transformed into (9S,13S)-12-oxo-phytodienoic acid (OPDA) by the action of two mitochondrial enzymes, allene oxide-synthase (AOS) and allene oxide-cyclase (AOC). OPDA-OPR3 mediates the reduction of OPDA to 3-oxo-2-(cis-2′-pentenyl)-cyclopentane-1-octanoic acid (OPC-8:0), a reaction that occurs in the peroxisomes. OPC-8:0 is the precursor of JA-Ile, the most bioactive JA (Wang et al., 2021). JA-Ile initiates the signaling cascade that activates a range of defense-related genes, ultimately promoting the biosynthesis of SMs.

Our study showed that exogenous MeJA positively regulated several genes encoding jasmonate-ZIM (zinc finger inflorescence meristem, JAZ) domain proteins (*JAZ5, JAZ6*, and *JAZ8*). JAZ proteins are essential regulators of plant growth and stress responses (Zhao et al., 2024). The degradation of JAZ proteins is an indispensable step in the release of MYC2*)*, a central regulator of the JA signaling pathway, which has been detected in *T. peruviana*. MYC2 is also involved in the regulation of various physiological processes and the biosynthesis of specialized metabolites in plants (Chung et al., 2008; Sohn et al., 2022). Therefore, the enhancement of JA signaling provides a plausible explanation for the increased accumulation of TPC, TFv, and TCG in MeJA-treated *T. peruviana* cultures.

It is important to clarify that, under our experimental conditions, the expression of some JA biosynthesis transcripts (such as AOS and AOC) did not show significant differences in MeJA-treated cultures. This could be explained by a time lag between the expression of these genes (early response) and the peak of SMs accumulation in *T. peruviana*. Consequently, one of the recommendations of this study is to monitor gene expression at multiple elicitation time points.

### 4.4 MeJA-induced shift in oxidative status in *T. peruviana*

Another documented effect of exogenous MeJA in plants is the induction of both enzymatic and non-enzymatic antioxidant defense systems. In our study, exogenous MeJA activated the expression of genes encoding antioxidant enzymes, such as *POD, POD4, POD5*, and *HAD*. *POD*s are involved in lignification and the scavenging of reactive oxygen species (ROS), thus protecting plant cells from oxidative stress induced by pathogens or mechanical damage (Passardi et al., 2004); In plant cell cultures, mechanical damage serves as an important source of ROS which can affect cell viability as, culture time progresses. HADs (haloacid dehalogenase-like hydrolase) are another group of enzymes that can mitigate ROS-induced damage by enhancing the activities of POD and superoxide dismutase (SOD) (Zan et al., 2023).

MeJA-mediated responses also include the upregulation of transcripts from the cytochrome family (*CYP450, CYP92A5*). CYPs participate in various biosynthetic pathways and detoxification reactions, conferring protection against biotic and abiotic factors (Zan et al., 2023). Previous studies have shown that the upregulation of some members of the CYP450 superfamily promotes flavonoid biosynthesis (Ahmad et al., 2019), as well as the accumulation of lignin and p-coumaric acid derivatives (Renault et al., 2017). Additionally, Ralston et al., 2001 associated CYP92A5 expression with an enhanced elicitor response in *Nicotiana tabacum* cell cultures.

Antioxidant strategies mediated by PCs and PODs have been previously described in *T. peruviana* cell cultures at the 7-liter bioreactor scale (Arias et al., 2021). An early response to ROS was observed, primarily mediated by antioxidant metabolites such as PCs and Fvs. This was followed by an enzyme-mediated response, where *PODs* and other antioxidant enzymes played a crucial role in ROS detoxification, particularly after seven days of culture. The results presented here contribute to our understanding of the antioxidant mechanisms induced by MeJA in *T. peruviana* cells cultured *in vitro*. It is confirmed that MeJA induces changes in cellular oxidative status through the expression of antioxidant enzyme genes (*PODs, HA, SOD*, and *CPYs*), which contribute to mitigating the cytotoxic effect of *ROS* in these cultures.

### 4.5 Transcriptional regulation of MeJA-mediated phenylpropanoids biosynthesis in *T. peruviana*

Exogenous MeJA induced the upregulation of genes in the phenylpropanoid pathway overexpressed in *T. peruviana* cells treated with MeJA, correlating with the increased accumulation of TPC and TFv. Notably, the upregulation of shikimate O-hydroxycinnamoyltransferase (*HST)*, a key gen in lignin biosynthesis, aligns with the increased accumulation of cinnamates, such as chlorogenic acid (CGA) and ferulic acid (FA), observed in *T. peruviana* cell cultures treated with MeJA (Mendoza et al., 2019). *HST* catalyzes the esterification of hydroxycinnamic-acids using shikimic acid or quinic acid as acyl acceptors, while also using cinnamoyl-CoA and p-coumaroyl CoA as acyl donors. Consequently, *HST* plays a crucial role in the biosynthesis of both caffeoyl shikimic acid and chlorogenic acid (CGA) (Liu et al., 2024). Additionally, CoA derivatives serve as precursors for flavonoid biosynthesis via chalcone intermediates (Kalariya et al., 2024). The preferential carbon flux towards PCs and Fvs biosynthesis was further supported by the downregulation of the *CCR1* (cinnamoyl CoA-reductase) transcript in *T. peruviana* cells treated with MeJA (**Figure 7**).

Hydroxycinnamoyl transferase (SHT) is another transcript of the phenylpropanoid pathway overexpressed in *T. peruviana* cells treated with MeJA. The role of SHT in flavonoid biosynthesis in *A. thaliana* was previously demonstrated by Besseau et al., 2007. By silencing the *SHT* gene, they observed repression in lignin synthesis and redirection of metabolic flux towards flavonoid biosynthesis, likely through the activation of chalcone synthase (*HICH*). It is possible that the expression of other key genes involved in Fvs biosynthesis occurs before 96 h post-elicitation (the time at which the highest TFv accumulation was recorded). Future studies will aim to evaluate the effect of elicitation time on the differential expression of these genes.

MeJA also led to the upregulation of the *FLS/F3H* (flavonol synthase/flavanone 3-hydroxylase) gene, which encodes an enzyme that catalyzes the conversion of naringenin into dihydroflavonols, followed by their oxidation to flavonols. Specifically, flavonol synthase (*FLS*) activity converts dihydrokaempferol (DHK) to kaempferol and dihydroquercetin (DHQ) to quercetin, contributing to flavonol biosynthesis in *T. peruviana* (Tewtrakul et al., 2002).

Additionally, MeJA treatment resulted in downregulation of DFR1, a transcript which encodes a key enzyme in the biosynthesis of anthocyanins and proanthocyanidins. DFR1 catalyzes the reduction of dihydroflavonols (e.g. DHK, DHQ) into their corresponding leucoanthocyanidins, a highly regulated step in plants that serves as a metabolic control point for anthocyanidin pathway flux (Sun et al., 2021b). The observed decrease in DFR1 expression aligns with the increased accumulation of flavonols in *T. peruviana* cultures treated with MeJA. Furthermore, the induction of FaGT6 (flavonol 3-O-glucosyltransferase) transcript by MeJA promotes flavonol glycosylation (Gao et al., 2023), potentially enhancing flavonol solubility, stability, and bioactivity.

### 4.6 Transcriptional regulation of MeJA-mediated cardiac glycosides biosynthesis in *T. peruviana*

Our results demonstrate that MeJA significantly increased CGs accumulation in *T. peruviana* throughout almost the entire treatment period. Consistent with this, transcriptomic analysis confirmed that MeJA upregulates the expression of genes involved in the biosynthesis of CGs precursors, such as dimethyl allyl pyrophosphate (*DMAPP*) and isopentenyl-diphosphate (*IPP*), which are produced through two distinct metabolic pathways: the mevalonate and methylerythritol phosphate (*MEP*) pathways (George et al., 2015). Furthermore, the direction of carbon flow toward the biosynthesis of these metabolic precursors was favored by the upregulation of key enzymes in energy metabolism, such as *HKL3* (hexokinase-like 3), *PFK3* (phosphofructokinase 3), *GAPDH3* (glyceraldehyde-3-phosphate dehydrogenase) and *PK* (cytosolic pyruvate kinase), along with the low expression of genes involved in primary anabolic reactions, such as the biosynthesis of polysaccharides and proteins.

Among the transcripts promoting the biosynthesis of CGs precursors are: ISPF (2-C-methyl-D-erythritol 2,4-cyclodiphosphate synthase, chloroplastic), TPS (terpenoid synthase), SQS1 (squalene synthase 1), IPP2 (Isopentenyl-diphosphate Delta-isomerase II) and CYP710A3. (**Supplementary Table S4**). Additionally, a transcript encoding 3-oxo-5-alpha-steroid 4-dehydrogenase (NADP+) was also detected in *T. peruviana*. These enzymes catalyze the conversion of various 3-oxo-delta-4 steroids into their corresponding 5-alpha-3-oxosteroids, a reaction previously described in the CGs biosynthetic pathway of *Calotropis procera (* Pandey et al., 2016 *)*. Gene expression analysis also revealed that MeJA upregulates candidate genes for the *SCL14* and *DWF1* proteins, which promote sterol and CGs biosynthesis (Amiri et al., 2023).

It is important to highlight that elucidating the complete CGs biosynthetic pathway remains a challenge, as several of the reactions involved are still unknown. Therefore, additional molecular studies are needed to provide more detailed information about this pathway and facilitate the identification of genes regulated by JAs.

### 4.7 Exogenous MeJA influences the expression of a specific set of transcription factors involved in PCs, Fvs and CGs biosynthesis

Among the TFs regulated by MeJA are proteins containing basic helix-loop-helix domain (bHLH18 and bHLH93), MYB, bZIP (basic leucine zipper domain), ethylene response factors (ERFs), and multiple members of the WRKY family. These TFs can regulate the expression of various structural genes in secondary biosynthetic pathways, acting downstream of JA signaling.

The bHLH family is one of the most important and abundant groups of TFs in plants. They are characterized by a basic DNA-binding domain followed by two alpha-helices separated by a variable loop region. A subset of JA-responsive TFs, known as MYC, contains conserved bHLH domains that allow DNA binding through the basic region, thereby activating responses to oxidative stress (Yoon et al., 2020). In our study, MeJA-treated cells exhibited increased expression of several bHLH proteins (including bHLH93, bHLH18 and bHLH13). It has been reported that bHLH13 and bHLH18 significantly enhanced *POD*, *SOD*, and catalase activity in plants subjected to abiotic stress (Liu et al., 2024; Zhang et al., 2024). Similarly, in *T. peruviana*, MeJA treatment may activate anti-ROS mechanisms through the expression of bHLH-regulated antioxidant enzymes, thus protecting cells from oxidative damage during *in vitro* culture.

Additionally, bHLH93 has been reported to play a significant role in the positive regulation of phenylpropanoid metabolism. A study by Jin et al. (2024) demonstrated that bHLH93 along with other TFs of the MYB family, induces the expression of Fvs biosynthesis genes and other PCs in jujube fruits (*Ziziphus jujuba Mil*l.). Specifically, MYB41 and bHLH93 directly bind to the promoters of genes encoding PAL and dihydroflavonol 4-reductase (*DFR*), thereby activating their transcription. Furthermore, bHLH93 is implicated in the catabolism of gibberellic acid (GA), a hormone known to suppress Fvs biosynthesis and regulate various aspects of plant growth and development (Sun et al., 2021a).

Consequently, our findings suggest that bHLH93 may play a key role in regulating the expression of structural genes involved in the biosynthesis of PCs and Fvs in *T. peruviana*. However, further molecular studies are needed to validate the structural and functional relationship between bHLH93 and the MeJA-positively regulated genes *HST, SHT, HICH* and *FLS/F3H*.

MeJA-induced MYB proteins constitute another group of TFs that regulate the expression of genes involved in SMs biosynthesis. The role of MeJA-responsive MYBs has been studied in *Salvia miltiorrhiza*, where their upregulation significantly enhances the accumulation of phenolic acids and positively regulates the expression of genes encoding key enzymes in their biosynthetic pathway (Zhou et al., 2021). Similarly, a study in buckwheat (*Fagopyrum tataricum*) confirmed the role of MYB102, a transcription factor upregulated in *T. peruviana* cells treated with MeJA, in the accumulation of the flavonoid rutin. Moreover, FtMYB102 was shown to interact with bHLH proteins, forming a transcriptional complex that binds to the chalcone isomerase (CHI) promoter, thereby promoting rutin biosynthesis (Mi et al., 2023). The formation of bHLH - R2R3-MYB transcriptional activator complexes, which regulate phenylpropanoid metabolism, has also been described in *Arabidopsis* (Petroni and Tonelli, 2011) ​. Given that MYB102 and bHLH transcripts were both upregulated by MeJA in *T. peruviana*, it is possible that the activation of Fvs and PCs biosynthesis in these species follows a mechanism like that observed in *F. tataricum* and *Arabidopsis*.

Like MYB and bHLH, proteins of the WRKY and bZIP families are TFs involved in abiotic stress tolerance and SM regulation in various plant species. JA-induced WRKYs have been linked to the biosynthesis of phenylpropanoids (Sohn et al., 2022) ​ and triterpenoids (Wen et al., 2023)​. Members of this WRKY family (e.g. WRKY53, WRKY70 and WRKY72) specifically bind to the W box (5’-(T)TGAC[CT]-3’), a cis-acting element present in the promoter sequences of target genes, thereby regulating their expression. WRKY72 has been identified as a transcriptional activator of terpene biosynthesis in glandular trichomes of *Solanum lycopersicum*, where it binds to the linalool synthase promoter and activates TPS5 gene transcription (Spyropoulou et al., 2014)​. It is proposed that the increase expression of WRKY and bZIP TFs in *T. peruviana* may be associated with enhanced CGs accumulation in MeJA-treated cells, potentially through a mechanism like that documented in S*. lycopersicum*.

Additionally, there is substantial evidence that members of the bZIP family regulate carbon and nitrogen metabolism. In *Ginkgo biloba* L., the elicitors MeJA and SA induced the expression of bZIP proteins involved in flavonoid biosynthesis in a time-dependent manner (Han et al., 2021)​. The bZIP44 transcription factor, induced by exogenous MeJA in *T. peruviana*, has also been identified as a key regulator of abscisic acid (ABA)-mediated carotenoid biosynthesis in *Citrus* spp. The regulatory mechanisms of bZIP44 involve both its direct binding to promoters of carotenoid metabolism genes and its interaction with a homeobox-leucine zipper protein (HB5), forming the CsHB5-CsbZIP44 transcriptional regulatory module. This module responds to ABA signaling and promotes carotenoid accumulation in citrus (Sun et al., 2024).

MeJA also influenced the regulation of ethylene (ET) signaling. ET signaling pathways have been extensively studied in plants, as they constitute a complex network of synergistic and antagonistic interactions that regulate stress responses, including SMs biosynthesis. In our study, treatment of *T. peruviana* cells with MeJA led to the upregulation of certain ERF transcripts, while others were downregulated. One of the MeJA-induced transcripts in *T. peruviana* was *ERF.C.3* that acts downstream of MYC2 in JA-mediated responses to pathogen infection in *S. lycopersicum.* However, this response was not associated to SMs accumulation (Du et al., 2017).

Our findings offer new insights into the regulation of TFs such as bHLH, MYB, bZIP and WRKY, following exogenous MeJA treatment. The expression of these TFs likely contributes to the activation of genes involved in the biosynthesis of SMs of pharmaceutical value (e.g. peruvoside, thevetin, flavanones and specific flavanols) in *T. peruviana* cells cultured *in vitro*.

## 5 Conclusion

In this study, we present the first *de novo* transcriptome of *T. peruviana*, marking a significant advancement in the genomic exploration of this medicinally valuable species. Using high-throughput RNA sequencing, we generated over 520 million high-quality reads, enabling the assembly of a comprehensive dataset comprising 83,126 unigenes. The high N50 value (3570 bp) and extensive functional annotation across key protein databases (NR, Uniprot, Pfam, KOG) underscore the depth and reliability of the assembly. This transcriptome offers a solid and versatile foundation for future research, providing valuable insights into the genetic architecture of *T. peruviana* and paving the way for a deeper understanding of its metabolic potential.

Furthermore, differential gene expression analysis reveals that exogenous MeJA promotes JA biosynthesis and signaling, along with the activation of MYC2-dependent TFs, processes that play a crucial role in the biosynthesis of PCs, Fvs, and CGs in *T. peruviana* cells. Additionally, MeJA treatment altered the oxidative status of *T. peruviana*, inducing the expression of antioxidant enzymes (e.g., POD, HAD, SOD, and CPY450) and antioxidant metabolites (TPC and TFv), thereby helping to protect the cells from ROS-induced damage during *in vitro* culture.

The results also revealed that MeJA induces a significant metabolic reprogramming in *T. peruviana* cells, characterized by the upregulation of energy metabolism genes (e.g., HKL3, PFK3, GAPDH3, and PK) that supply metabolic precursors for the biosynthesis of phenylpropanoid (such as cinnamates and flavonoids) and CGs. Concurrently, MeJA downregulated genes involved in polysaccharide and protein biosynthesis. These transcriptome changes were consistent with the increased accumulation of PCs, Fvs, and CGs observed in *T. peruviana* suspension cells at 96 hours post-elicitation.

A total of 22 transcripts related to sterol and CGs biosynthesis were identified, several of which were significantly upregulated in MeJA-treated cells (e.g., ISPF, TPS, SQS1, IPP2, CYP710A3, SCL14, DWF1). However, no transcripts associated with downstream reactions of 5-alpha-3-oxosteroid, specific to the CGs biosynthetic pathway, were detected. Given that many enzymatic steps within this pathway remain uncharacterized, our findings represent a meaningful advancement in elucidating CGs biosynthesis in *T. peruviana*.

Finally, although our data reveals strong transcriptomic response 96 h post-MeJA elicitation (2,587 upregulated and 2,482 downregulated genes), we acknowledge the importance of assessing the temporal lag between gene expression and metabolite accumulation in future studies. Such time-resolved analysis would enable a more accurate characterization of gene expression dynamics associated with secondary metabolite biosynthesis. This, in turn, would deepen our understanding of the regulatory networks involved and support the development of targeted metabolic engineering strategies to enhance the production of pharmaceutically valuable compounds (e.g. peruvoside, thevetins, flavanones, and flavonols) in *T. peruviana* suspension cell cultures.

## Data Availability

The data presented in the study are deposited in the NCBI Sequence Read Archive (SRA), accession numbers SAMN42862923 to SAMN42862930 within BioProject PRJNA1140843. https://www.ncbi.nlm.nih.gov/bioproject/PRJNA1140843 The assembly fasta file and annotation files are available in figshare at https://doi.org/10.6084/m9.figshare.26764642.v1.

## Supplementary material

Supplementary Figure 1 Pipeline for transcriptome *de novo* assembly and annotation.

Supplementary Figure 2 MDS plot based on expression profiles.

Supplementary Figure 3 GO Terms of transcripts with differential expression.

Supplementary Table 1 Supplementary materials and methods: colorimetric technical and assembly *de novo*.

Supplementary Table 2 Summary of sequencing reads data.

Supplementary Table 3 Sequencing of primers to qPCR.

Supplementary Table 4 Differential expressed anotated gens.

Supplementary Table 5 Top 10 differentially expressed up and down genes.

Supplementary Table 6 Differential expressed genes of secondary metabolism.

Supplementary Table 7 Annotated transcriptional factors.
