# Supplementary material for: Transcriptome analysis of Thevetia peruviana cell suspensions treated with methyl jasmonate reveals genes involved in phenolics, flavonoids and cardiac glycosides biosynthesis
Source: Front Plant Sci. 2025 May 26;16:1593315. doi: 10.3389/fpls.2025.1593315 (PMC12146404; doi:10.3389/fpls.2025.1593315)

Supplementary Material

**Figure S1. Bioinformatics pipeline for transcriptome de novo assembly and annotation.**


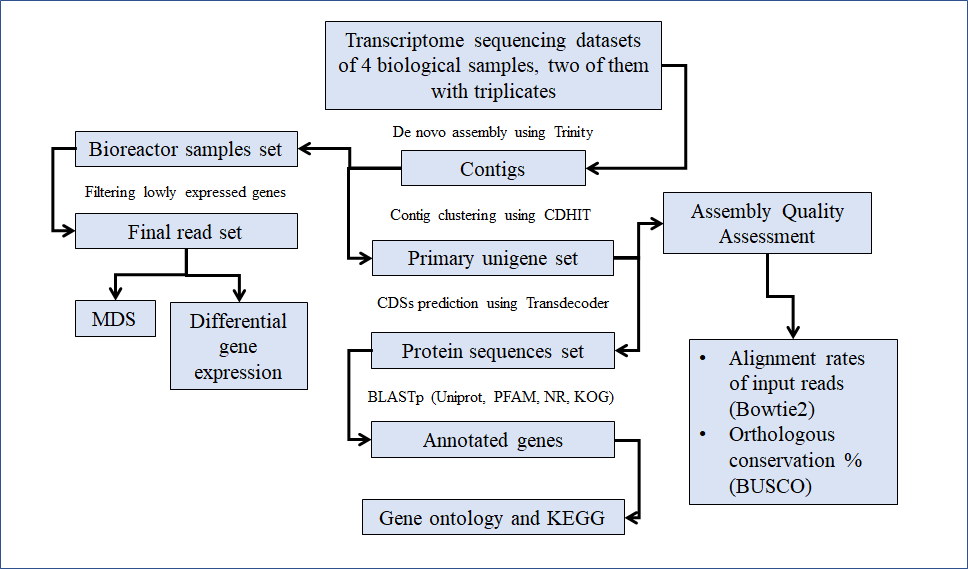

Supplement: Supplementary Figure 1 — Pipeline for transcriptome de novo assembly and annotation. [file Table1.docx]
