# Supplementary material for: Transcriptome analysis of Thevetia peruviana cell suspensions treated with methyl jasmonate reveals genes involved in phenolics, flavonoids and cardiac glycosides biosynthesis
Source: Front Plant Sci. 2025 May 26;16:1593315. doi: 10.3389/fpls.2025.1593315 (PMC12146404; doi:10.3389/fpls.2025.1593315)

Supplementary Material

**Figure S2. MDS Plot Based on Expression Profiles**. Clustering of samples reflects their source of origin. Dimension 1 (not shown) separates samples from natural plants (leaves and roots) from suspension cell samples (MeJA (ST) colored purple and elicited with water (SC) colored yellow). Component 2 distinguishes leaf samples from root samples and suspension cell samples, while Dimension 3 separates suspension cell samples elicited with MeJA (ST) from those elicited with water (SC).


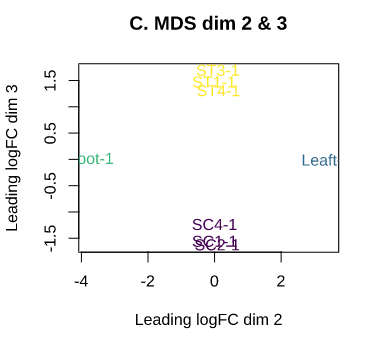

Supplement: Supplementary Figure 2 — MDS plot based on expression profiles. [file Table2.docx]
