# Supplementary material for: Transcriptome analysis of Thevetia peruviana cell suspensions treated with methyl jasmonate reveals genes involved in phenolics, flavonoids and cardiac glycosides biosynthesis
Source: Front Plant Sci. 2025 May 26;16:1593315. doi: 10.3389/fpls.2025.1593315 (PMC12146404; doi:10.3389/fpls.2025.1593315)

Supplementary Material

**Figure S3. GO Terms of Transcripts with Differential Expression.** The bar graphs represent the number of differentially expressed transcripts annotated to respective biological process GO terms. The results for downregulated genes in treated samples are shown on the left in blue, while the results for upregulated genes in treated samples are shown on the right in red. A single gene may be assigned to multiple GO terms according to the hierarchy of the terms; here, only the first three levels of annotation are presented.


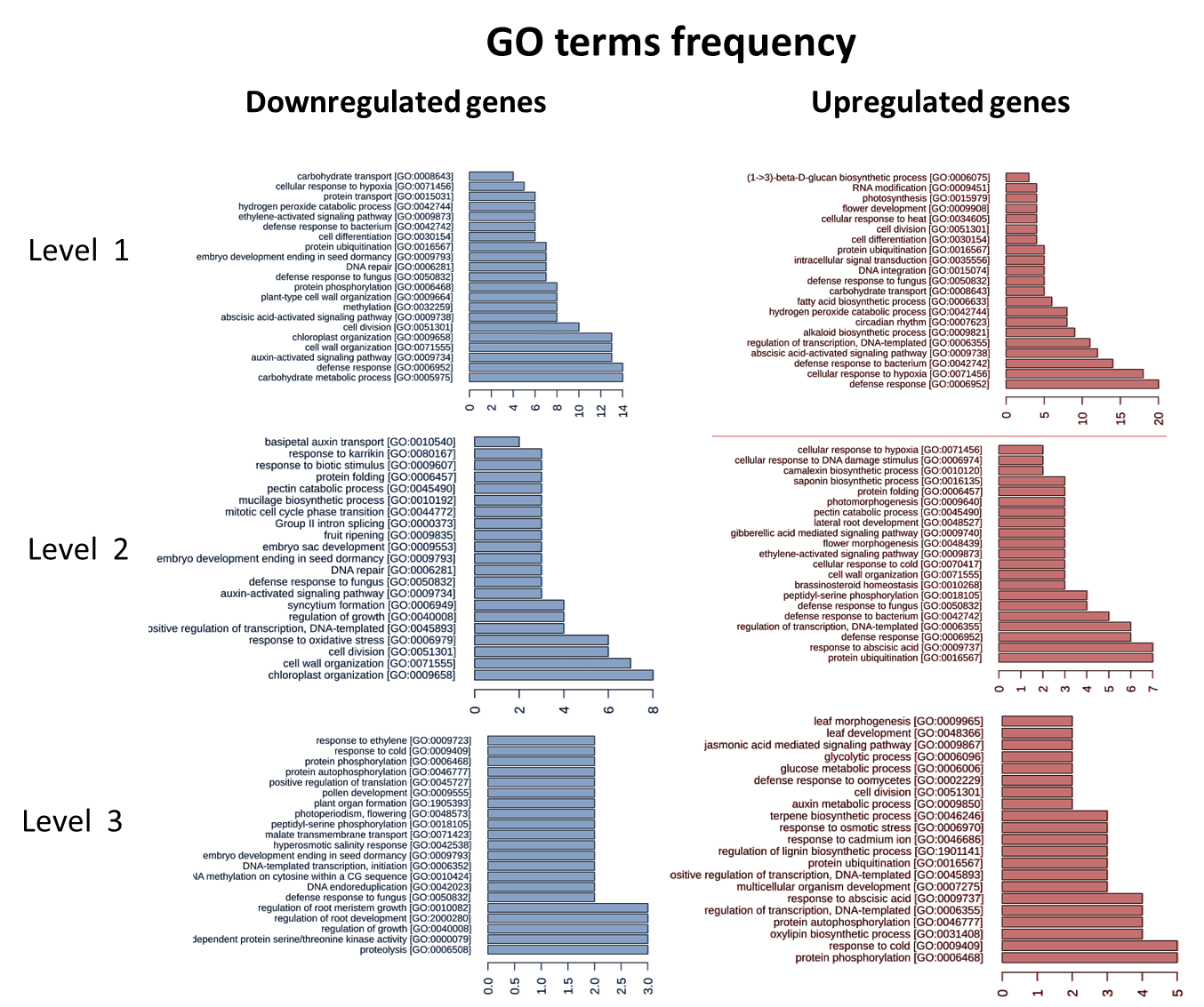

Supplement: Supplementary Figure 3 — GO Terms of transcripts with differential expression. [file Table3.docx]
