# Supplementary material for: Transcriptome analysis of Thevetia peruviana cell suspensions treated with methyl jasmonate reveals genes involved in phenolics, flavonoids and cardiac glycosides biosynthesis
Source: Front Plant Sci. 2025 May 26;16:1593315. doi: 10.3389/fpls.2025.1593315 (PMC12146404; doi:10.3389/fpls.2025.1593315)
Supplement: Supplementary Table 1 — Supplementary materials and methods: colorimetric technical and assembly de novo. [file Table4.docx]

**Supplementary Material**

**Table S1 - Supplementary materials and methods**

| **Total Phenolic compounds (TPC)** |
| --- |
| TPC were determined using the Folin-Ciocalteu method (Lantzouraki et al., 2015), with gallic acid (GA) as the standard. A 15 µl aliquot of the sample was mixed with 37 µl of 10 % (v/v) Folin-Ciocalteu reagent and 128 µl of distilled water. After 2 minutes, 120 µl of 7.5 % (p/v) Na_2_CO_3_ were added, followed by incubation at 50 °C for 10 minutes. Absorbance was measured at 765 nm using a Multiskan Spectrophotometer (Thermo Fisher Scientific®). Intracellular TPC was quantified using a gallic acid standard curve in the range of 0.63 to 40.0 mg/mL (R² = 0.990), prepared by serial two-fold dilutions. TPC was expressed as milligrams of gallic acid equivalent per gram of dry weight (mg GAE/g DW). |
| **Total Flavonoids (TFv)** |
| TFv content was quantified using a colorimetric method (Zou et al., 2011). A 30 μl aliquot of the sample solution was mixed with 180 μL of distilled water in a well of a 96-well plate. Subsequently, 10 μL of a 5 % NaNO_2_ solution was added. After 6 minutes, 20 μL of a 10 % AlCl_3_ solution was introduced to the mixture and left to stand for 15 minutes. Absorbance was measured at 510 nm using quercetin (QE) as the standard (4-500 µg/mL, R² = 0.992). All results were expressed as milligrams of quercetin equivalent per gram of dry weight (mg QE/g DW). |
| **Total Cardiac Glycosides (TCG)** |
| TCG were determined using a modified Baljet method (Tofighi et al., 2016) in 96-well plates. A 75 μL aliquote of the sample solution was mixed with 75 μL of Baljet reagent (95 mL of 1% picric acid + 5 mL of 10% NaOH) and incubated in the dark for 1 hour. Subsequently, 150 μL of distilled water was added and absorbance was measured at 495 nm. The reference curve was constructed using Digoxin (DE) as the standard (6.25 - 250 mg/L), and results were expressed as milligrams of digoxin equivalents per gram of dry weight (mg DE/g DW). |
| **Total Triterpenoid (TTp)** |
| TTp content was assessed using a modified colorimetric method described by Chang & Lin (2012) for a 96 well plate format. Briefly, 10μL of the sample was mixed with 15 μL of a 5 % (w/v) vanillin/glacial acetic acid solution, followed by the addition of 50 μL of a 70 % (v/v) perchloric acid solution. The mixture was incubated at 60 °C for 45 minutes in an oven, then cooled in an ice bath until reaching 28°C. Subsequently, 225 μL of glacial acetic acid was added. Finally, absorbance was measured at 548 nm. The reference curve was constructed using ursolic acid (0.04 - 2.5 mg/mL), and the results were expressed as milligrams of ursolic acid equivalents per gram of dry weight (mg UAE/g DW). |
| **Transcriptome assembly** |
| ***Pre-assembly quality control and filtering.***  The sequencing data underwent quality assessment using the FastQC software version 0.11.9 (Andrews S., 2010). The initial analysis confirmed excellent overall quality (Q >20) reported by BGI; however, non-random frequency of nitrogenous bases was observed within the first 19 positions, alongside 5 to 20 % duplication of sequences. To rectify these issues, the Trimommatic software v.0.39 (Bolger et al., 2014) was employed to remove the first 19 bases from all sequences. Subsequent confirmation of the cleaning process's efficiency was achieved through rerunning FastQC.  The SILVA ribosomal RNA database Version 138.1 (Glöckner et al., 2017) was employed to verify the absence of rRNA contamination. This involved acquiring SSUParc and LSUParc fasta files, concatenating them, and replacing U with T. Subsequently, reads were mapped to the database using Bowtie2 software version 2.4.4 (Langmead et al., 2019). Results indicated no evidence of bacterial contamination.  Despite efforts to eliminate duplicate reads using the PrinSeq software version 0.20.4 (R & R, 2011), the persistence of duplicate reads was noted, attributed to limitations in handling non-exact duplicates. While we have addressed the most significant concerns in data cleaning, we acknowledge the possibility of other factors, such as highly expressed genomic regions or sequences derived from repetitive elements contributing to observed duplication.  ***De novo transcriptome assembly.***  Since this is the first time that the transcriptome of *T. peruvian*a has been sequenced and its genome has not been published before either, a *de novo* approach was used for this transcriptome assembly. For this purpose, the software Trinity v2.12.0 with default k-mer size = 25 bp (Grabherr et al., 2011) was employed. Trinity has been considered a standard in de novo assembly protocols (Chabikwa et al., 2020; Chen et al., 2024; Kang et al., 2024; Raghavan et al., 2022; Xie et al., 2024) due to its broad user community and consistently high performance at default k-mer size (25 pb), comparable to other leading assemblers (Hölzer & Marz, 2019; Liu et al., 2016). Since its release in 2011, this tool has been cited over 16,000 times (Raghavan et al., 2022), making it a trustworthy choice for assembly. K mer size was kept as default (25 pb) also to match the k mer size employed by the large-scale effort “One thousand plant transcriptomes Initiative” (Leebens-Mack et al., 2019).  **General Information of Assembly**   \| **Assembly Stats** \| **based on ALL Contigs** \| **based on LONGEST ISOFORM per 'GENE'** \| \| --- \| --- \| --- \| \| Contig N10 \| 7243 \| 6731 \| \| Contig N20 \| 5790 \| 5206 \| \| Contig N30 \| 4870 \| 4197 \| \| Contig N40 \| 4167 \| 3364 \| \| Contig N50 \| 3570 \| 2547 \| \| Median contig length \| 1063 \| 376 \| \| Average contig \| 1867 \| 936 \| \| **Total assembled bases** \| **362636488** \| **93517630** \|   **Post-assembly quality control**  The quality of the assembly was evaluated following standard guidelines for assembly assessment (Raghavan et al., 2022), focusing on read support, defined as the fraction of total reads mapping back to the assembly (>80%). Clean reads from each sample were mapped to the assembled transcriptome using Bowtie2 software version 2.4.4 (Langmead & Salzberg, 2012). The assembly was further examined for the presence of orthologs of genes universal to the eudicot lineage. Representation of conserved genes was assessed using BUSCO version 5.2.2 (Simão et al., 2015), employing the eudicot_odb10 dataset, which includes 2326 orthologs from 31 genomes. To minimize redundancy within the assembly, thinning was performed using the clustering tool CD-HIT version 4.8.1 (W & A, 2006). Representative sequences, termed 'unigenes,' were identified from each cluster as those exhibiting the highest similarity to other cluster members, sharing over 95% identity.  Alignment results of the clean reads to the Trinity assembly showed that over 98% of the sequenced reads from each tissue were successfully mapped, indicating a high degree of completeness and representativeness of the de novo assembled transcriptome   \| **Sample** \| **Label** \| **Total clean reads** \| **Percentage of aligned reads** \| \| --- \| --- \| --- \| --- \| \| Leaf \| Leaf \| 34207553 \| 98.51% \| \| Root \| Root \| 35187728 \| 98.52% \| \| Control \| Ctrl1 \| 33826818 \| 98.31% \| \| Control \| Ctrl2 \| 33810257 \| 98.38% \| \| Control \| Ctrl3 \| 33680239 \| 98.52% \| \| Treatment \| MeJA1 \| 30509309 \| 98.38% \| \| Treatment \| MeJA2 \| 34870168 \| 98.24% \| \| Treatment \| MeJA3 \| 33919859 \| 98.43% \| |
| ***De novo* transcriptome assembly.** |
| Open reading frames from assembled unigenes were predicted and translated using Transdecoder distributed alongside Trinity. The 102 824 predicted aminoacid sequences were queried against the Nr (Sayers et al., 2022), Uni-Prot (UNIPROT, 2000), Pfam (EMBL-EBI, 2021) and KOG (National Center for Biotechnology Information, 2020) and Clusters of Eukaryotic Orthologous Groups (KOG) (National Center for Biotechnology Information, 2020) databases using the BLASTp software (Camacho et al., 2009) (e <0,00001 y pident> 50%).  All assembled unigenes were annotated by BLASTp analysis against the Nr (Sayers et al., 2022), Uni-Prot (UNIPROT, 2000), Pfam (EMBL-EBI, 2021) and KOG (National Center for Biotechnology Information, 2020) databases with an e-value <1e-5. Only the top hit results were extracted for each unigene. Gene Ontology (Carbon et al., 2021) terms were functionally classified based on Nr annotations. The analysis results included KEGG orthology (Kanehisa et al., 2007) numbers and enzyme commission (EC) numbers.  **BLASTp alignment results of Trinity-assembled sequences against various databases for transcriptome annotation**   \| **Database** \| **Hits** \| **Annotated peptides** \| **min pident** \| **max pident** \| **mean pident** \| **Hits with pident >50** \| **Annotated peptides with pident >50** \| **Unique DB entries with pident >50** \| \| --- \| --- \| --- \| --- \| --- \| --- \| --- \| --- \| --- \| \| PFAM \| 101173 \| 93891 \| 18.00 \| 100 \| 60.99 \| 72266 \| 57561 \| 57561 \| \| KOG \| 98944 \| 90708 \| 18.68 \| 100 \| 59.04 \| 67243 \| 52883 \| 52883 \| \| UNIPROT \| 90 95 \| 82913 \| 18.58 \| 100 \| 58.00 \| 57050 \| 56267 \| 45441 \| \| NR NCBI \| 12164 \| 8490 \| 19.89 \| 100 \| 34.77 \| 755 \| 749 \| 618 \|   pident=percentage of identity |

**References**

Andrews, S. (2010). FastQC. Available at: https://www.bioinformatics.babraham.ac.uk/index.html

Bolger, A. M., Lohse, M., and Usadel, B. (2014). Trimmomatic: a flexible trimmer for Illumina sequence data. *Bioinformatics* 30, 2114–2120. doi: 10.1093/bioinformatics/btu170

Carbon, S., Douglass, E., Good, B. M., Unni, D. R., Harris, N. L., Mungall, C. J., et al. (2021). The Gene Ontology resource: enriching a GOld mine. *Nucleic Acids Res* 49, D325–D334. doi: 10.1093/nar/gkaa1113

Chabikwa, T. G., Barbier, F. F., Tanurdzic, M., and Beveridge, C. A. (2020). De novo transcriptome assembly and annotation for gene discovery in avocado, macadamia and mango. *Sci Data* 7, 9. doi: 10.1038/s41597-019-0350-9

Chang, C. L., and Lin, C. S. (2012). Phytochemical Composition, Antioxidant Activity, and Neuroprotective Effect of Terminalia chebula Retzius Extracts. *Evidence-Based Complementary and Alternative Medicine* 2012, 1–7. doi: 10.1155/2012/125247

Chen, L., Hu, Y., Huang, L., Chen, L., Duan, X., Wang, G., et al. (2024). Comparative transcriptome revealed the molecular responses of Aconitum carmichaelii Debx. to downy mildew at different stages of disease development. *BMC Plant Biol* 24, 332. doi: 10.1186/s12870-024-05048-x

EMBL-EBI (2021). Database Pfam. *Pfam 35.0 (November 2021, 19632 entries)*. Available at: pfam.xfam.org

Glöckner, F. O., Yilmaz, P., Quast, C., Gerken, J., Beccati, A., Ciuprina, A., et al. (2017). 25 years of serving the community with ribosomal RNA gene reference databases and tools. *J Biotechnol* 261, 169–176. doi: 10.1016/j.jbiotec.2017.06.1198

Kanehisa, M., Araki, M., Goto, S., Hattori, M., Hirakawa, M., Itoh, M., et al. (2007). KEGG for linking genomes to life and the environment. *Nucleic Acids Res* 36, D480–D484. doi: 10.1093/nar/gkm882

Kang, J.-N., Hur, M., Kim, C.-K., Yang, S.-H., and Lee, S.-M. (2024). Enhancing transcriptome analysis in medicinal plants: multiple unigene sets in Astragalus membranaceus. *Front Plant Sci* 15. doi: 10.3389/fpls.2024.1301526

Langmead, B., and Salzberg, S. L. (2012). Fast gapped-read alignment with Bowtie 2. *Nat Methods* 9, 357–359. doi: 10.1038/nmeth.1923

Langmead, B., Wilks, C., Antonescu, V., and Charles, R. (2019). Scaling read aligners to hundreds of threads on general-purpose processors. *Bioinformatics* 35, 421–432. doi: 10.1093/bioinformatics/bty648

Lantzouraki, D. Z., Sinanoglou, V. J., Tsiaka, T., Proestos, C., and Zoumpoulakis, P. (2015). Total phenolic content, antioxidant capacity and phytochemical profiling of grape and pomegranate wines. *RSC Adv* 5, 101683–101692. doi: 10.1039/C5RA20064D

Li, W., and Godzik, A. (2006). Cd-hit: a fast program for clustering and comparing large sets of protein or nucleotide sequences. *Bioinformatics* 22, 1658–1659. doi: 10.1093/bioinformatics/btl158

Raghavan, V., Kraft, L., Mesny, F., and Rigerte, L. (2022). A simple guide to *de novo* transcriptome assembly and annotation. *Brief Bioinform* 23. doi: 10.1093/bib/bbab563

Sayers, E. W., Bolton, E. E., Brister, J. R., Canese, K., Chan, J., Comeau, D. C., et al. (2022). Database resources of the national center for biotechnology information. *Nucleic Acids Res* 50, D20–D26. doi: 10.1093/nar/gkab1112

Schmieder, R., and Edwards, R. (2011). Quality control and preprocessing of metagenomic datasets. *Bioinformatics* 27, 863–864. doi: 10.1093/bioinformatics/btr026

Simão, F. A., Waterhouse, R. M., Ioannidis, P., Kriventseva, E. V., and Zdobnov, E. M. (2015). BUSCO: assessing genome assembly and annotation completeness with single-copy orthologs. *Bioinformatics* 31, 3210–3212. doi: 10.1093/bioinformatics/btv351

UNIPROT (2000). Database Uniprot ref. *Uniprotref*. Available at: https://www.uniprot.org/downloads

Xie, Q., Deng, W., Su, Y., Ma, L., Yang, H., Yao, F., et al. (2024). Transcriptome Analysis Reveals Novel Insights into the Hyperaccumulator Phytolacca acinosa Roxb. Responses to Cadmium Stress. *Plants* 13, 297. doi: 10.3390/plants13020297

Zou, Y., Chang, S. K. C., Gu, Y., and Qian, S. Y. (2011). Antioxidant Activity and Phenolic Compositions of Lentil (Lens culinaris var. Morton) Extract and Its Fractions. *J Agric Food Chem* 59, 2268–2276. doi: 10.1021/jf104640k
