# Supplementary material for: Transcriptome analysis of Thevetia peruviana cell suspensions treated with methyl jasmonate reveals genes involved in phenolics, flavonoids and cardiac glycosides biosynthesis
Source: Front Plant Sci. 2025 May 26;16:1593315. doi: 10.3389/fpls.2025.1593315 (PMC12146404; doi:10.3389/fpls.2025.1593315)
Supplement: Supplementary Table 2 — Summary of sequencing reads data. [file Table5.docx]

Supplementary Material

**Table S2. Summary of sequencing reads data metrics provided by BGI.**

| **Sample Name** | **Clean Reads** | **Clean bases** | **Read Length (pb)** | **Q20(%)** | **GC(%)** |
| --- | --- | --- | --- | --- | --- |
| **Leaft-1** | 68.415.106 | 10.262.265.900 | 150 | 96,18% | 44,18% |
| **Root-1** | 70.375.456 | 10.556.318.400 | 150 | 95,74% | 44,06% |
| **SC1-1** | 67.653.636 | 10.148.045.400 | 150 | 96,04% | 44,33% |
| **SC2-1** | 67.620.514 | 10.143.077.100 | 150 | 96,59% | 44,24% |
| **SC4-1** | 67.360.478 | 10.104.071.700 | 150 | 96,61% | 44,07% |
| **ST1-1** | 61.018.618 | 9.152.792.700 | 150 | 95,97% | 44,00% |
| **ST3-1** | 69.740.336 | 10.461.050.400 | 150 | 96,17% | 43,98% |
| **ST4-1** | 67.839.718 | 10.175.957.700 | 150 | 96,64% | 43,94% |

*ST: Sample Treated

*SC: Sample Control
