# Supplementary material for: Transcriptome analysis of Thevetia peruviana cell suspensions treated with methyl jasmonate reveals genes involved in phenolics, flavonoids and cardiac glycosides biosynthesis
Source: Front Plant Sci. 2025 May 26;16:1593315. doi: 10.3389/fpls.2025.1593315 (PMC12146404; doi:10.3389/fpls.2025.1593315)
Supplement: Supplementary Table 5 — Top 10 differentially expressed up and down genes. [file Table8.docx]

**
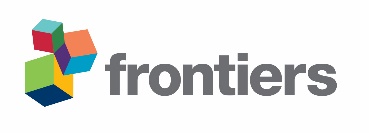
**

**Supplementary Material**

**Table S5 - Top 10 differentially expressed up and down genes**

Top 10 differentially expressed genes (Up and Down) between cell suspension samples treated with 3 µM MeJA and control samples.

| **Log2(FC)** | **p-value** | **Gene** | **Protein name** | **Process** |
| --- | --- | --- | --- | --- |
| **4.79** | 0.0004 | TRINITY_DN831_c4_g1 | UDP9: Glycosyltransferase 9 | Involved in metabolic processes; enables UDP-glucosyltransferase activity |
| **4.75** | 0.0077 | TRINITY_DN8693_c0_g1 | GL1: Trichome differentiation protein | Encodes GL1, a Myb-like protein that is required for induction of trichome development. Interacts with JAZ and DELLA proteins to regulate trichome initiation. |
| **4.72** | 0.0004 | TRINITY_DN17042_c0_g1 | UDP9: Glycosyltransferase 9 | Involved in metabolic processes; enables UDP-glucosyltransferase activity |
| **4.65** | 0.0037 | TRINITY_DN6492_C0-G1 | BHlH18: Transcription factor | Involved in regulation of DNA-templated transcription |
| **4.40** | 0.0357 | TRINITY_DN5305_C0_G1 | RG1: Raucaffricine-O-beta-D-glucosidase | Enables beta-glucosidase activity; involved_in carbohydrate metabolic process. |
| **4.33** | 0.0003 | TRINITY_DN6948_c0_g1 | RBOHD: Respiratory burst oxidase | Acts upstream of or within respiratory burst involved in defense response. |
| **4.30** | 0.0037 | TRINITY_DN3698_C0_G2 | LS: Lupeol synthase | Acts upstream of or within pentacyclic triterpenoid biosynthetic; involved in triterpenoid biosynthetic process |
| **3.98** | 0.00116 | TRINITY_DN10146_c0_g1 | IQM1: IQ domain-containing protein | Involved in the modulation of stomatal movement. Promotes stomatal opening. May play a role in the regulation of chitin signaling. May be involved in biotic and abiotic stress responses |
| **3.91** | 0.0013 | TRINITY_DN68028_c0_g1 | HST: O-hydroxycinnamoyl transferase | Acyltransferase involved in the biosynthesis of lignin; Accepts caffeoyl-CoA and p-coumaroyl-CoA as substrates and transfers the acyl group on both shikimate and quinate acceptors |
| **3.88** | 0.0010 | TRINITY_DN82592_c0_g1 | mRNA, 952 bp sequence | - |
| **- 4.97** | 0.0019 | TRINITY_DN6063_c0_g1 | RD21B: Probable cysteine protease | Involved in proteolysis involved in protein catabolic process |
| **- 5.15** | 0.0016 | TRINITY_DN25980_c0_g1 | ERF-LEP: Ethylene-responsive transcription factor LEP | Involved in regulation of DNA-templated transcription |
| **- 5.76** | 0.0007 | TRINITY_DN5194_c0_g1 | RGI2: LRR receptor-like serine/threonine- PK | Acts as a receptor of RGF peptides, peptide hormones which maintain the postembryonic root stem cell niche by regulating the expression levels and patterns of the transcription factor PLETHORA |
| **- 5.80** | 0.0014 | TRINITY_DN15327_c0_g1 | At1g77230 | Uncharacterized protein |
| **- 5.81** | 0.0003 | TRINITY_DN1888_c0_g2 | Early nodulin-93 (N-93) | Enables molecular function, essential for induction of somatic embryogenesis. |
| **- 6.35** | 0.0025 | TRINITY_DN36616_c0_g1 | ALEU: Thiol protease | Involved in proteolysis involved in protein catabolic process. |
| **- 6.39** | 0.0021 | TRINITY_DN2057_c0_g1 | PIN: Auxin efflux carrier component | Involved in auxin export across the plasma membrane; acts upstream of or within somatic embryogenesis. |
| **- 6.99** | 0.0029 | TRINITY_DN9669_c1_g1 | PG: Polygalacturonase | Involved in carbohydrate metabolic process |
| **- 7.82** | 0.0012 | TRINITY_DN9669_c1_g2 | PG Polygalacturonase | Involved in carbohydrate metabolic process. |
| **- 8.46** | 0.0013 | TRINITY_DN1875_c0_g1 | GH3.6: Indole-3 acetic acid-amido synthetase | Enables indole-3-acetic acid amido synthetase activity. |
